# Genetic and environmental modulation of transposition shapes the evolutionary potential of *Arabidopsis thaliana*

**DOI:** 10.1186/s13059-021-02348-5

**Published:** 2021-05-06

**Authors:** Pierre Baduel, Basile Leduque, Amandine Ignace, Isabelle Gy, José Gil, Olivier Loudet, Vincent Colot, Leandro Quadrana

**Affiliations:** 1 Institut de Biologie de l’École Normale Supérieure, ENS, 46 rue d’Ulm, 75005 Paris, France; 2 Institut Jean-Pierre Bourgin, INRAE, AgroParisTech, Université Paris-Saclay, 78000 Versailles, France; 3 Present Address: Institut Curie, 26 rue d’Ulm, 75005 Paris, France

**Keywords:** Transposable elements, Genome evolution, Population genetics, Epigenomics, Adaptation, Climate change

## Abstract

**Background:**

How species can adapt to abrupt environmental changes, particularly in the absence of standing genetic variation, is poorly understood and a pressing question in the face of ongoing climate change. Here we leverage publicly available multi-omic and bio-climatic data for more than 1000 wild *Arabidopsis thaliana* accessions to determine the rate of transposable element (TE) mobilization and its potential to create adaptive variation in natural settings.

**Results:**

We demonstrate that TE insertions arise at almost the same rate as base substitutions. Mobilization activity of individual TE families varies greatly between accessions, in association with genetic and environmental factors as well as through complex gene-environment interactions. Although the distribution of TE insertions across the genome is ultimately shaped by purifying selection, reflecting their typically strong deleterious effects when located near or within genes, numerous recent TE-containing alleles show signatures of positive selection. Moreover, high rates of transposition appear positively selected at the edge of the species’ ecological niche. Based on these findings, we predict through mathematical modeling higher transposition activity in Mediterranean regions within the next decades in response to global warming, which in turn should accelerate the creation of large-effect alleles.

**Conclusions:**

Our study reveals that TE mobilization is a major generator of genetic variation in *A. thaliana* that is finely modulated by genetic and environmental factors. These findings and modeling indicate that TEs may be essential genomic players in the demise or rescue of native populations in times of climate crises.

## Background

Adaptation to rapidly changing environments in the absence of standing genetic variation is a long-standing genetic paradox [1, 2]. Indeed, mutations typically arise at low rates and produce neutral variants predominantly. However, this picture ignores sequence alterations generated by the mobilization of transposable elements (TEs), which have many properties that distinguish them from “classical,” small-size mutations. First, TEs are powerful endogenous mutagens: through their mobilization, they can disrupt or alter genes as well as their expression in multiple ways, and because of their dispersion across the genome, they provide many opportunities for the creation of chromosomal rearrangements through ectopic recombination [3, 4].

Eukaryotic TEs belong to two broad classes: DNA transposons, which use a cut and paste mechanism for their mobilization, and retrotransposons, which move through an RNA intermediate [5]. These two classes are further divided into TE superfamilies and families based on particular sequence features, such as the presence or absence of long terminal repeats (LTRs) in the case of retrotransposons [5].

Population genomic surveys of TE insertion polymorphisms (TIPs) revealed that many TEs insert preferentially towards genes and that insertions are rapidly purged from gene-rich regions [6, 7], suggesting that natural transposition tends to generate alleles with strong deleterious effects. Epigenetic mechanisms, which include DNA methylation in plants and animals, have evolved to limit TE mobilization. In plants, DNA methylation of TE sequences encompasses the three cytosine contexts (CG, CHG, and CHH, where H is A, T, or C). In the reference plant *Arabidopsis thaliana*, establishment of DNA methylation at TEs occurs in an RNA-dependent manner (RNA-directed DNA methylation or RdDM) and requires the activity of the de novo DNA methyltransferases DRM1/2 as well as of two plant-specific RNA Pol II derivatives, Pol IV and Pol V. TE methylation is then maintained through replication by the DNA methyltransferases CMT3 and MET1, which act respectively on CHGs and CGs, as well as by DRM1/2 and CMT2, which have mostly non-overlapping CHH targets [8]. DNA methylation deficiencies do not lead by themselves to widespread TE re-mobilization [9–14], indicating that additional factors control transposition. For instance, mobilization in the Col-0 background of the LTR-retroelement *ONSEN*, which belongs to the *ATCOPIA78* family, was shown experimentally to require heat-shock in addition to impaired RdDM activity [15], thus demonstrating that at least in this case, both genetic and environmental determinants are decisive.

Although there is evidence of sustained transposition activity in *A. thaliana* [6, 16], a comprehensive understanding of the factors involved is missing. Here, we leveraged the sampling depth of the *A. thaliana* 1001 Genomes project (1001genomes.org [17];) to identify the major factors associated with recent TE mobilization and to determine the impact of the thousands of insertions near or within genes it generated. We then use ecological modeling to explore the evolutionary trajectories resulting from this recent activity and to predict the consequences of global warming on the creation of genetic variation through transposition in the near future.

## Results

### Recent TE mobilization at the species level

**Fig. 1:**
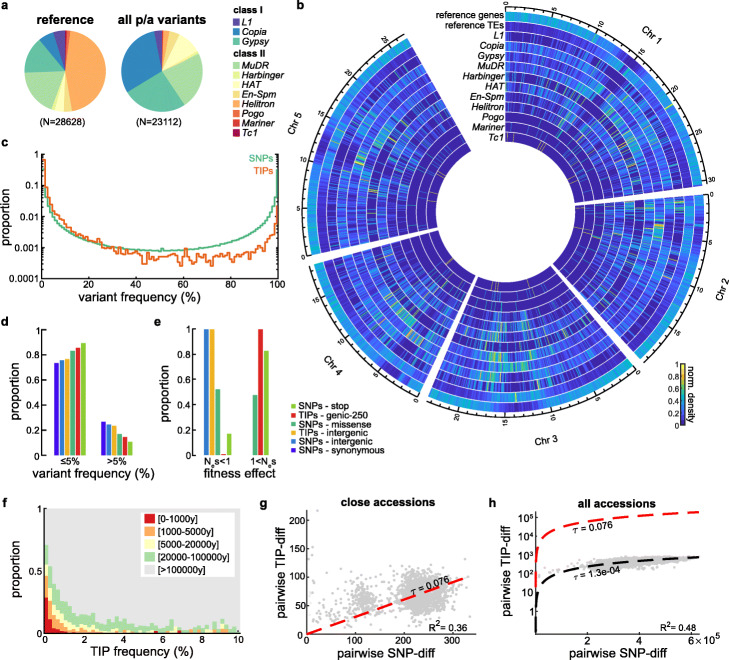
Recent TE mobilization at the species level. **a** Relative proportion of the 11 major TE superfamilies identified in the reference genome (TAIR10) and their respective contribution to TE insertion polymorphisms (TIPs). **b** Density of TIPs across the genome for each superfamily compared to the distribution of genes or TEs annotated in the reference genome. **c** Folded frequency spectrum of TIPs and biallelic SNPs. **d** Proportion of each variant category at frequencies below 5% and above 5%. **e** Distribution of fitness effects of each variant category as effectively neutral (N_e_s < 1) and deleterious (1 < N_e_s). **f** Frequency distribution of non-private TIPs by local haplotype age. **g** Pairwise differences in TIPs and SNPs between accessions diverging by < 500 SNPs. Regression line and confidence intervals are indicated in red and gray, respectively. **h** Pairwise differences in TIPs and SNPs between all accessions. Regression lines between all and closely related accessions are shown in black and red, respectively

In order to evaluate recent transposition dynamics in *A. thaliana*, we used short-reads sequencing data available for 1047 Arabidopsis accessions of the 1001 Genomes project (1001genomes.org, Additional file 1: Table S1) and searched for TE insertion polymorphisms (TIPs). TIPs were identified using a bioinformatic pipeline [18] combining SPLITREADER [6] and TEPID [16], which efficiently detect the presence of non-reference TE sequences and the absence of reference TE sequences in resequenced genomes, respectively. After stringent filtering (see the “Materials and methods” section), we recovered 23,331 high-confidence TIPs, including 21,707 non-reference TE presence variants (Additional file 2: Dataset S1). These were contributed almost entirely by the two superfamilies of LTR retrotransposons *COPIA* and *GYPSY* (respectively 6941 and 5794 TIPs) and the two superfamilies of DNA transposons *MuDR* and *hAT* (respectively 4973 and 2101 TIPs, Fig. 1a). Presence variants for the DNA transposon *HELITRON* superfamily were ignored as they are not efficiently detected by our pipeline, unlike the corresponding absence variants, which together with *MuDR* and *hAT* make up over half of the 1624 absence variants detected in total (Fig. 1a; Additional file 3: Fig. S1a). TIPs are broadly distributed across the genome, with the notable exception of those produced by the *GYPSY* superfamily of LTR retrotransposons, which are enriched in pericentromeric regions. The broad distribution of TIPs confirms previous observations obtained using a smaller number of non-reference genomes [6] and is in stark contrast with the relative paucity of reference TE sequences along the chromosome arms and their high density in pericentromeric regions (Fig. 1b). Furthermore, the site frequency spectrum (SFS) of TIPs, which we calculated using the number of informative genomes at each site, is heavily skewed towards low values compared to biallelic SNPs (Fig. 1c). Specifically, one third of TIPs have a minor allele frequency of less than 0.2% and > 80% of these were missed in previous analyses based on ∼200 genomes [6]. The excess of low-frequency TIPs compared to SNPs suggests strong negative effects. Indeed, TE insertions located near (< 250 bp) or within genes are present at frequencies similar to that of missense SNPs (Fig. 1d,e; Additional file 3: Fig. S1b). To estimate how deleterious TE insertions are, we computed the distribution of fitness effects (DFE) of each category of variants by comparing their SFS with that of synonymous SNPs to control for recent demographic changes (DFE-alpha; [19]) as they can affect SFSs in ways that resemble selection. Using this approach, we estimate that > 99% of TE insertions within 250 bp of a gene are deleterious (N_e_s > 1), compared to 48% and 83% of missense and nonsense SNPs, respectively (Fig. 1e). Thus, almost all TE insertions within or adjacent to genes are associated with sizable deleterious effects.

Most low-frequency TIPs should reflect recent transposition events not yet purged by natural selection [6, 20–22]. To determine the relationship between age and TIP frequency, we considered all TIPs shared by at least two genomes and estimated their age by first calculating for each TE insertion the number of SNPs accumulated in its vicinity (35 kb on either side; see “Materials and methods”). We then transformed this number into a predicted age by applying the base mutation rate of 7E-9 per genome per year determined experimentally [23] and ignoring the possibility of a slight increase in mutation rates locally (within 3 kb) following TE insertion, as was reported for rice and other grasses [24]. Using this approach, we found a positive correlation between predicted age and TIP frequency (*R*^2^ = 0.4; Additional file 3: Fig. S1c). However, this result indicates that TIP frequency is not a perfect proxy for age as only half of TIPs that segregate at frequencies below 1.5% are less than 5000 years old (Fig. 1f).

We next estimated the substitution rate for TE insertions using closely related accessions (i.e., accessions that differ by < 500 SNPs, Additional file 3: Fig. S1d) and found it to be almost a third (0.076 +/− 0.0012 per genome per generation; Fig. 1g, see the “Materials and methods” section) of that calculated for base substitutions [25]. In contrast, the most divergent accessions differ by a maximum of ~ 730 TIPs, which is two orders of magnitude lower than expected if TIPs were to accumulate at the same rate as between closely related accessions (Fig. 1h). Indeed, we predict (see the “Materials and methods” section) that > 99.8% of TE insertions that occur in nature are eventually eliminated by natural selection, a percentage higher than for missense and even nonsense SNPs (68.9% and 92.5%, respectively; Additional file 3: Fig. S1d-e). Moreover, TE insertion substitutions within or near genes occur at rates ten-fold higher than that of nonsense and of the same order to that of missense base substitutions (0.025 vs 0.002 and 0.038 mutations per genome per generation, respectively; Additional file 3: Fig. S2a,d,e). Together, these results indicate that TE mobilization is a major contributor of large-effect genetic variants in *A. thaliana*.

### Genetic basis of variable transposition

**Fig. 2:**
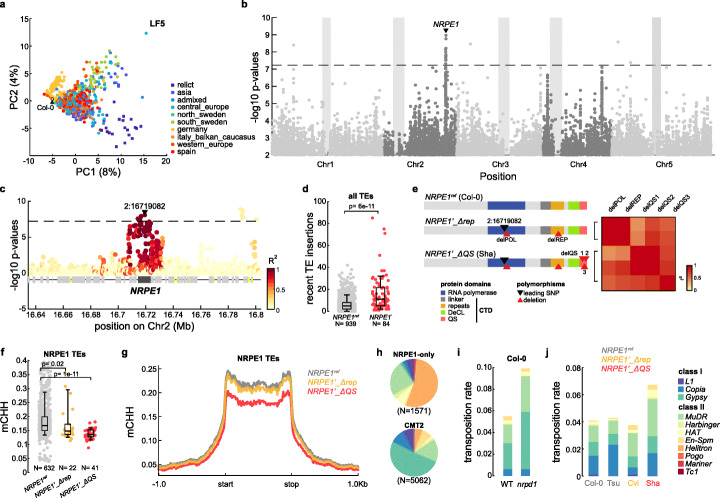
Genetic basis of variable transposition. **a** PCA of mobilome composition based on very recent TIPs (MAF lower than 5%). Different genetic groups are indicated in colors. **b** Manhattan plot of GWAS for very recent genome-wide TE mobilization. Dashed line represents the Bonferroni-corrected threshold for significance. **c** Detailed Manhattan plot within 80 kb around *NRPE1* locus. Colors indicate the extent of linkage disequilibrium (*R*^2^) with the leading SNP (black triangle). **d** Boxplot of numbers of very recent TE insertions in carriers of the reference *NRPE1*^*ref*^ and derived *NRPE1’* alleles. The *p* value of a Wilcoxon test between distributions is indicated. **e** Alleles and polymorphisms at *NRPE1* locus and the linkage between their closest tagging SNPs. **f, g** Boxplot and metaplot of CHH methylation on *NRPE1-*dependent TEs within carriers of the derived *NRPE1’_ΔQS* allele, carriers of the derived *NRPE1’_Δrep* allele, and a set of 100 randomly sampled carriers of the reference *NRPE1*^*ref*^ allele. The *p* values of Wilcoxon tests between distributions are indicated. **h** Composition by superfamily of *NRPE1- or CMT2-*targeted TE sequences. **i** Transposition rates in 1000 offspring of WT and *nrpd1* parents of the Col-0 accession grown under standard conditions. **j** Transposition rates in 1000 offspring of the Cvi-0, Sha, Tsu-0, and Col-0 accessions derived from parents grown under standard conditions

To explore further the mutation pressure associated with TE mobilization in nature, we first carried out principal component analysis using the number of TIPs with a MAF ≤ 5% per TE family. Results revealed a significant structuration of overall transposition activity in relation to the 10 main genetic groups defined in *A. thaliana* [26], with Relicts, Asian and South-Sweden accessions being the most contrasted (Fig. 2a; Additional file 3: Fig. S3a).

Based on this finding, we searched for potential genetic modifiers of global transposition activity. To this end, we performed a genome-wide association study (GWAS) using as a quantitative trait the total number of very recent TE insertions per genome across all TE families (TE insertions with MAF lower than 0.2% and < 1000 years old or private; Additional file 3: Fig. S3b-c). This analysis differs therefore from our previous analysis aimed at identifying genetic factors associated with copy number variation at the single TE family level [6]. These GWASs revealed strong *cis*-associations, as expected, as well as several shared *trans*-associations, the most common of which involved the poorly characterized *MET2a* gene and a disparate group of seven TE families [6]. In contrast, our new study revealed a single major peak of *trans*-association with recent TE mobilization (Fig. 2b), suggesting a simple genetic architecture of global transposition activity. Furthermore, this association peak spans the gene *NUCLEAR RNA POLYMERASE E1* (*NRPE1;* Fig. 2c), which encodes the largest subunit of RNA Pol V, an essential component of RdDM [27] and was previously identified in GWAS as a major determinant of CHH methylation at TEs targeted by RdDM [28]. Moreover, the non-reference allele, called *NRPE1’*, which is linked to reduced CHH methylation, is associated with a 240% increase in transposition activity (Fig. 2d), thus strongly supporting a causal role. GWAS performed at the TE superfamily level revealed in addition that associations with *NRPE1* are strongest for *MuDRs*, which have RdDM- rather than CMT2-dependent CHH methylation, (Fig. 2h, see the “Materials and methods” section), effectively confirming causality (Additional file 3: Fig. S3e).

Inspection of long-read sequencing data [29] from an accession (Sha) carrying the *NRPE1’* allele revealed extra polymorphisms beyond the SNPs and short-indels identified by the 1001 genomes project [26]. Specifically, the *NRPE1’* allele of Sha contains also a 9-bp in-frame deletion in the region encoding the 17aa-repeat domain as well as three deletions in the region encoding the QS tail of the C-terminal domain (CTD; respectively 6 bp-, 60 bp-, and 9 bp-long deletions, Fig. 2e; Additional file 3: Fig. S3g). In fact, the three QS deletions (delQS1-2-3) of the CTD define a suballele of *NRPE1’*, which we named *NRPE1’_ΔQS* in contrast to *NRPE1’_Δrep* that carries the 17aa-repeat deletion and at most two of the three QS deletions (Fig. 2e; Additional file 3: Fig. S3g). Moreover, the two derived *NRPE1* alleles resemble those produced experimentally in the reference accession Col-0 [30] and they are associated with similar effects on CHH methylation of RdDM TE targets, with a more pronounced loss when the QS and repeat domains are deleted together (Fig. 2f,g; Additional file 3: Fig. S3f). Remarkably, the two naturally truncated alleles explain by themselves at least 17% of the variation in transposition activity at the species level (Additional file 3: Fig. S3d, Additional file 4: Table S2, see below).

To evaluate directly the impact of impaired RdDM on overall transposition activity, we carried out TE sequence capture [9] on pools of 1000 seedlings of parents of the Col-0 reference accession grown under standard conditions that are either WT or mutant for the gene *NRPD1* (Additional file 5: Dataset S2), which encodes the largest subunit of RNA Pol IV. A total 99 novel TE insertions were detected in the *nrpd1* sample (Fig. 2i), a 80% increase compared to the WT. Higher transposition in *nrpd1* was most prominent for *GYPSYs* and *MuDRs*, consistent with most of their CHH methylation being RdDM-dependent, unlike that of *COPIAs*, which are targeted by CMT2 also (Additional file 3: Fig. S3h). Furthermore, the rate of transposition determined experimentally is of the same order of the substitution rate for TE insertions we estimated at the species level (0.06 in WT vs 0.08 per genome per generation, respectively), thus providing direct experimental support for the latter.

Using the same approach, we also measured TE mobilization in three natural accessions (Additional file 6: Dataset S3), including Cvi and Sha, which carry the *NRPE1’_Δrep* and *NRPE1’_ΔQS* alleles, respectively. Sha exhibited the highest transposition rate overall (Fig. 2j), which was mainly driven by *GYPSYs* and *MuDRs* (Additional file 3: Fig. S3i), thus resembling in this respect the *nrpd1* mutant of the reference accession Col-0. Even though numerous genetic variants segregate between these accessions, this observation is consistent with *NRPE1’* causing increased TE mobilization.

### Environmental modulation of TE mobilization

**Fig. 3:**
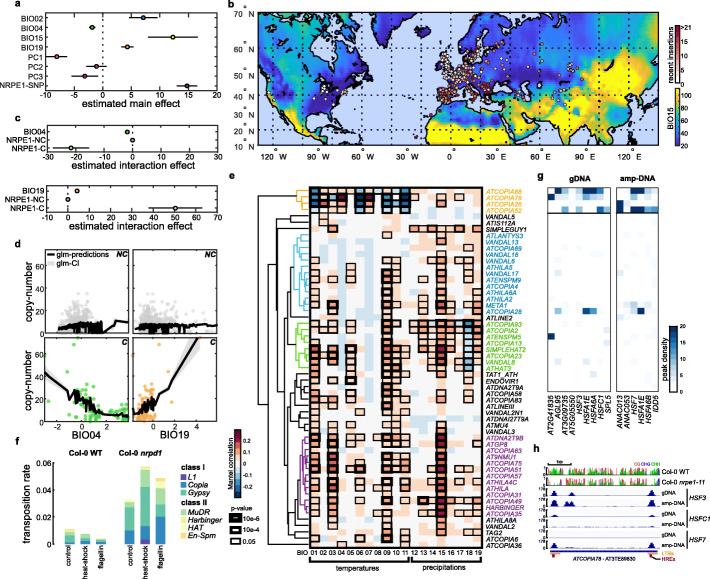
Environmental modulation of TE mobilization. **a** Marginal effect at the mean of each of the variables considered in the GLM of very recent transposition: the first three principal components of the kinship matrix (PC1-2-3), the *NRPE1* locus, and the BIO02, BIO04, BIO15, and BIO19 variables. **b** Number of very recent TE insertions detected across the world and levels of precipitation seasonality (BIO15). **c** Estimated interaction effect of BIO04 and *NRPE1* (upper) and BIO19 and *NRPE1* (bottom). **d** Scatter plot of very recent transposition against BIO04 (left) and BIO19 (right) in non-carriers (NC, up) and carriers (C, down) of the derived *NRPE1’* alleles. GLM predictions and confidence intervals are indicated in black and gray, respectively. **e** Directional Mantel associations for 77 TE families between very recent transposition and 19 WorldClim bio-variables (1970–2000) with dendrogram of hierarchical clustering of coefficient correlations. The four main clusters are indicated (colors). **f** Transposition rates in 1000 offspring of Col-0 WT and *nrpd1* parents grown under standard conditions or exposed to heat-shock or flagellin. **g** Normalized peak density of in vitro binding of TFs (DAP-seq) enriched over the “temperature” TE cluster in Col-0 gDNA and PCR-amplified DNA. **h** Tracks of DNA methylation (CG in red, CHG in blue, CHH in green) in Col-0 WT and *nrpe1_11* mutants and DAP-seq peaks of heat-shock factors HSF3, HSFC1, and HSF7 in Col-0 gDNA and PCR-amplified DNA*.* The position of the tandem heat-responsive elements (HREs: nTTCnnGAAn) [31] located in the LTRs are indicated in purple

We next investigated potential environmental modulators of transposition activity using 19 climatic bio-variables measured between the years 1970 and 2000 and which describe local patterns of temperature and precipitation variations (Worldclim.org). We performed a stepwise selection of the most relevant bio-variables on the basis of their added explanatory power in a generalized linear model (GLM) of very recent transposition that includes population structure and allelic variation at *NRPE1* (see the “Materials and methods” section). Importantly, given the previously described mobilization of *ONSEN *only when impairment of RdDM activity is combined with heat-shock [15], we considered in addition the possibility of GxE interactions involving *NRPE1* [15]. The GLM revealed that, while variation in transposition activity between accessions is explained predominantly (27%) by genetic backgrounds and allelic variation at *NRPE1*, seasonality of precipitation (BIO15) and diurnal temperature range (BIO02) explain another 9% of this variation (6.3% and 2.7%; Fig. 3a,d; Additional file 7: Table S3, Additional file 3: Fig. S4a). Furthermore, GxE interactions between *NRPE1’* and temperature seasonality and precipitation of the coldest quarter (BIO04 and BIO19 respectively; Fig. 3a,b) are also significant contributors, accounting for an additional 4.2% of variation in TE mobilization. In fact, differential TE mobilization in association with these two bio-variables is only observed for accessions carrying *NRPE1’* alleles (Fig. 3c,d), which extends to natural settings the experimental observation that mutations in the RdDM pathway modulate transposition in response to environmental changes.

To move beyond this global picture, we analyzed environmental associations at the TE family level using a Mantel test, which also incorporates population structure (see the “Materials and methods” section). Focusing on the 77 TE families with higher mobility and responsible for 89% of the very recent TIPs used for the GWAS and GLM analysis, we detected for 57 TE families significant associations with at least one environmental variable (Fig. 3e). Consistent with the GLM results, positive association with precipitation seasonality (BIO15) is most prevalent at the individual TE family level (44 out of 57 TE families; Fig. 3e). Moreover, we identified four clusters of TE families that share similar environmental associations. One small cluster of four *COPIA* TE families stands out by exhibiting the strongest associations, all of which concern temperature bio-variables. Consistent with previous work [6], *ATCOPIA78* belongs to this last cluster. The present analysis reveals in addition that the association of *ATCOPIA78* mobility with temperature is only observed in the *NRPE1’* background (Additional file 3: Fig. S4b-c), which mirrors the observation that transposition of the *ATCOPIA78* copy *ONSEN* present in Col-0 can only be induced following heat-shock in RdDM sensitized backgrounds [15].

To assess experimentally the extent of the interaction between RdDM and environmental stress, we compared transposition using TE sequence capture in pools of 1000 WT and *nrpd1* seedlings produced from Col-0 parents exposed this time to heat-shock or flagellin, a bacterial peptide known for triggering plant biotic stress response (see the “Materials and methods” section, Additional file 8: Dataset S4). Levels of TE mobilization in the progeny of WT parents grown under standard conditions appeared lower than in our two previous measurements (Fig. 3f), presumably as a result of differences in seed stocks and slight differences in growth conditions. Nonetheless, transposition clearly increased in the *nrpd1* mutant but not in WT following heat-shock (Fig. 3f), and this increase was not restricted to *ATCOPIA78 (ONSEN*) but concerned also most notably the *GYPSY* and *MuDR* families *ATGP1* and *VANDAL6*, respectively (Additional file 3: Fig. S4d). Similar but weaker *trans*-family sensitization by impaired RdDM was also observed following exposure to flagellin and concerned the same families in many cases, with the *COPIA* family *META1* being one notable exception that showed increased transposition only following flagellin treatment (Additional file 3: Fig. S4d).

To investigate the molecular underpinnings of these environmental responses, we re-mapped, including over TE sequences, in vitro DNA affinity purification sequencing (DAP-seq) datasets obtained in Col-0 using native or amplified (i.e., stripped of all DNA methylation) genomic DNA for 469 transcription factors (TFs) [32]. TE families in the three “precipitation” clusters share few enrichments for sites bound by specific TFs (TFBSs; Additional file 3: Fig. S5a), which suggests that their environmental responsiveness, notably to drought in the case of the cluster containing *ATCOPIA93* (Fig. S5h-i), can be acquired through a diverse set of transcriptional wirings. Indeed, the full-length, mobile copy of *ATCOPIA93* present in the Col-0 genome and known as *EVADÉ* (*EVD*) is transcriptionally inducible following exposure to biotic stress [33]. Thus, associations with drought may in fact result from activation by other stresses linked to precipitation variations. In contrast, the four *COPIA* TE families belonging to the “temperature” cluster share enrichments in TFBSs for 14 TFs (Fig. 3g). These TFs include six known heat-shock factors (HSF3, HSF7, HSFC1, HSFA1E, HSFA6A, and HSFA6B; Fig. 3g; Additional file 3: Fig. S5a-c) and another three TFs encoded by genes induced transcriptionally under heat-shock treatments (ANAC013, ANAC053, SPL5; Additional file 3: Fig. S5d-e, Additional file 9: Table S4). Even though *ATCOPIA28* does not belong to the temperature cluster, it appears similarly enriched for sites bound by HSFA1E, in agreement with its heat sensitivity [34]. Moreover, HSFA1s are essential for *ONSEN* induction upon heat-shock [31], consistent with the presence of tandem heat responsive elements (HREs) in its LTRs (Fig. 5h). Transcriptome data for the reference accession Col-0 indicate also that three of the four *COPIA* families in the temperature cluster are transcriptionally upregulated under heat-shock (Additional file 3: Fig. S5f-g), most prominently *ATCOPIA78*. Comparison of TF binding data on native genomic DNA as well as amplified DNA indicated that DNA methylation hinders the in vitro binding of HSF7, HSFA6B, and ANAC013 at these sites (Fig. 3e–g; Additional file 3: Fig. S5b-c), consistent with the sensitivity to DNA methylation reported for these TFs [32]. Finally, *ATCOPIA26*, which is not transcriptionally upregulated under heat-shock in Col-0, shows enrichment for the heat-responsive TF ANAC013 only when it is unmethylated (Fig. 3g). Together, these results point to an important role of environmentally responsive TFs and compromised DNA methylation in the increased mobilization of the temperature cluster of *COPIAs* that is observed in accessions carrying the *NRPE1’* derived alleles and exposed to extreme seasonal shifts in temperature.

### TE mobilization predominantly generates deleterious mutations within genes

**Fig. 4:**
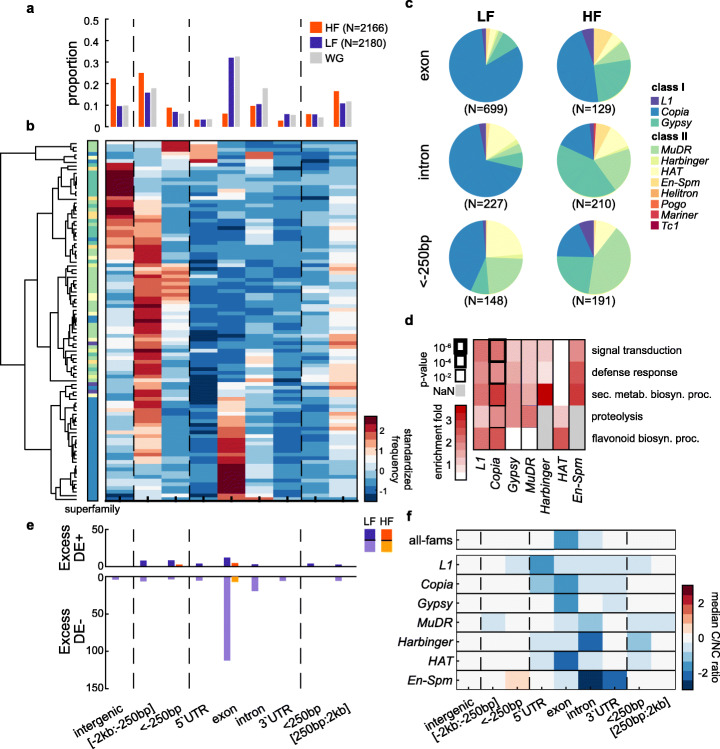
TE mobilization within or near genes predominantly generates deleterious mutations. **a** Fraction of low- and high-frequency TE presence variants overlapping genic annotations (exons, introns, 5′ or 3′ UTRs) or located near genes (upstream/downstream within 250 bp, or within 2 kb) or intergenic (> 2 kb away from nearest gene) compared to the genomic proportion of each category. **b** Insertion frequencies across genomic categories for TE families with ≥ 50 TIPs. Rows are standardized and clustered based on correlation distance. **c** Distribution of low- and high-frequency TE presence variants in exons, introns, and promoter regions (<− 250 bp) for each TE superfamily. **d** GO enrichments of LF presence variants within genes. **e** Excess of extreme expression log ratios between carriers (C) and non-carriers (NC) by insertion category at low- and high-frequency (negative = DE- at bottom, and positive = DE+ on top) compared to random sampling of carriers and non-carriers. **f** Median transcriptomic impact (C/NC expression ratio) by TE family by insertion category

To determine the mutation load generated by transposition, we measured the transcript levels of genes affected by the presence of TIPs near or within them. We ignored absence variants, as the presence of a TE annotation in the reference genome sequence at the corresponding position may have affected the annotation of the adjacent genes, thus complicating comparisons. In addition, we restricted our analysis to the rarest (first decile) TIPs present in one of at least 909 genomes, because collectively they provide the set of TIPs the least impacted by natural selection. Of the 2180 rarest non-reference TE presence variants (LF) retained for analysis, over 50% are located within genes, with exons being the most prevalent targets (66% of genic insertions, Fig. 4a) and as frequently hit as expected by chance. However, broad differences in insertion preferences can be observed across TE families, with *GYPSYs* found typically within intergenic regions, *MuDRs* within promoters (<− 250 bp) and 5′-UTRs and *COPIAs* within exons (Fig. 4b). As a result, the vast majority (> 70%) of exonic insertions are caused by *COPIAs* (Fig. 4c) and, consistent with experimental results [9], they affect preferentially environmentally responsive genes, especially those involved in defense response (Fig. 4d).

To assess the transcriptional impact of each of the 2180 LF non-reference TE insertions, we used matched (mature leaf before bolting) transcriptomes available for 604 of the 1047 accessions [35] and compared the average transcript level of the nearest gene in the TE-carrying accessions (C) to that in non-carrier (NC) accessions. As expected, most (~ 75%) TE insertions within exons are associated with reduced transcript levels (Fig. 4e) and almost all of these are contributed by *COPIAs* (111 out of 124; Additional file 3: Fig. S6a). Furthermore, in ~ 20% of cases, the TE-containing allele is an effective knock-out (Fig. S6b). TE insertions in introns are also frequently associated with reduced gene expression, but the effects are typically of smaller magnitude (Additional file 3: Fig. S6c). Despite these general trends, a few TE insertions are associated with increased transcript levels, and these are contributed mainly by *MuDRs* and tend to reside within the 5′ UTR or the promoter regions of genes (Fig. 4e; Additional file 3: Fig. S6a), consistent with the insertion preferences exhibited by this TE superfamily [9]. Altogether, these observations indicate that almost a quarter of mutations generated by TE mobilization in nature are likely to have major and mostly negative effects on gene transcript levels, with the remaining being either inconsequential or associated with increased expression in very rare cases.

To determine the evolutionary fate of the insertion mutations generated by TE mobilization in nature, we compared the genomic distribution of the 2180 LF TE-containing alleles with that of the 2166 most frequent (HF) ones (last decile, segregating at frequencies over 4.92%). In marked contrast to LF alleles, HF alleles are strongly biased away from genic sequences (~ 20% only vs ~ 60% expected based on the composition of genome, Fig. 4a). HF exonic insertions are particularly rare (5.9% of HF vs 32% of LF variants) and transcriptome data indicates that knock-out alleles are totally absent at high-frequency (Fig. 4e,f). Likewise, there are no HF intronic insertions associated with major reduction in gene expression (Fig. 4e; Additional file 3: Fig. S6c). Conversely, we recovered as many or more intronic or promoter variants at low and high frequency (respectively 227 vs 210 for introns and 148 vs 191 for the promoters; Fig. 4a,c), consistent with their minimal or positive transcriptomic impact, except in the case of *COPIA* insertions (Fig. 4e,f; Additional file 3: Fig. S6a). Whether any of the high-frequency insertions are under positive selection remains to be determined. Together, these findings confirm that the majority of TE insertions within or near genes are under strong purifying selection (Fig. 1e).

### Recurrent targeting of genes by TEs contributes to local adaptation

**Fig. 5:**
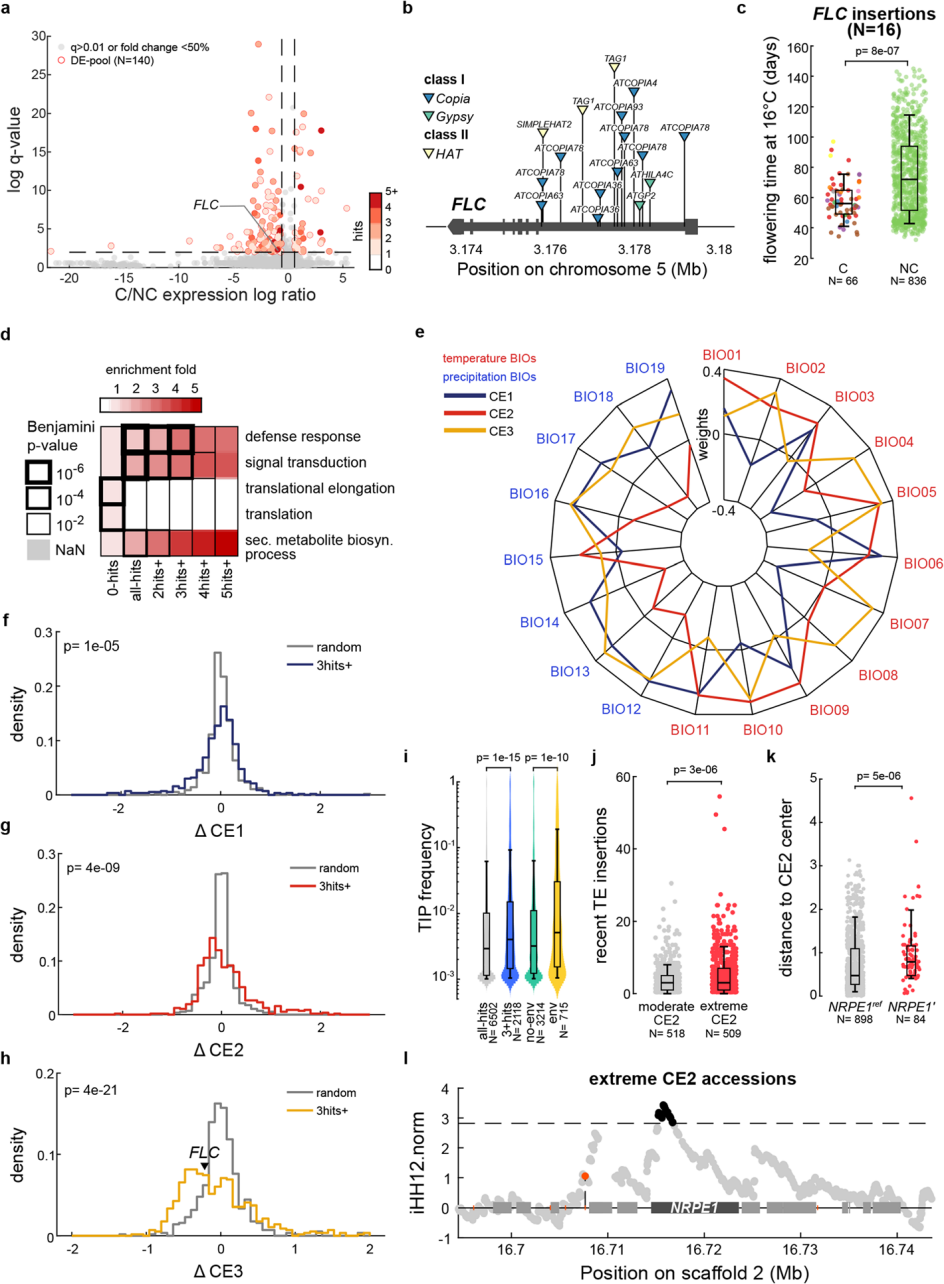
Contribution of transposition to local environmental adaptation. **a** Significance against log-ratio of combined transcriptomic effects of TE insertions within or near (< 250 bp) genes in carrier accessions (C) compared to non-carrier accessions (NC). The number of TE insertions found for each locus is indicated as a shade of red. **b** Location and identity of the 16 TE insertions detected within *FLC*. **c** Flowering time at 16 °C of carrier accessions (C) and non-carrier accessions (NC) of *FLC* TE-insertions. The *p* value of Wilcoxon test is indicated. **d** Top 5 GO enrichment terms across genes never visited or visited once or more. **e** Weights across 19 bio-variables of 3 first climatic envelopes (CEs) in PCA of 1047 accessions. **f–h** Distributions of climatic envelope shifts (ΔCEs) observed between carriers and non-carriers of TE insertions for each of the 566 genes hit 3 times or more compared to the distribution of ΔCEs with the same numbers of randomly selected carriers. The *p* values of Kolmogorov-Smirnov comparisons between observed and random distributions are indicated. **i** Frequency of TE insertions found within or near genes visited (all-hits), visited 3 times or more (3hits+), in association with a CE shift (env) or not (no-env). The *p* values of Wilcoxon tests between distributions are indicated. **j** Boxplot of numbers of very recent TE insertions in extreme or moderate CE2 accessions. The *p* values of Wilcoxon tests between distributions are indicated. **k** Boxplot of distance to CE2 center (absolute *z*-scored CE2) for carriers of the reference *NRPE1*^*ref*^ and derived *NRPE1’* alleles. The *p* values of Wilcoxon tests between distributions are indicated. **l** iHH12 values in extreme CE2 accessions (upper and lower quartiles of CE2.z) across the *NRPE1* region with in black indicated values above the genome-wide 1% threshold (dashed line)

Consistent with this last conclusion and because of the marked insertion preference of *COPIAs* towards responsive genes and away from essential genes [6, 9], the set of gene loci with TIPs is much smaller than expected by chance (4078 vs 9090 ± 45, see the “Materials and methods” section) and depleted in essential genes (Additional file 3: Fig. S7c). Conversely, TIP-containing gene loci with at least three distinct TE-containing alleles are more abundant than expected by chance (566 vs 285 ± 8, Fisher exact test *p* = 5e−51). As these alleles tend to be low-frequency variants, they could reflect either recurrent targeting because of insertion preferences, relaxed purifying selection, and/or diversifying selection. We can rule out an important role of insertion preferences, given the minimal overlap between gene loci visited in the lab and in nature for four TE families most active in these two settings (Additional file 3: Fig. S7a). Furthermore, the fact that pseudogenes are not strongly enriched in TE insertions (206 vs 167 expected by chance) indicates that multiple hits cannot solely result from relaxed purifying selection. Moreover, because 99% of gene loci with TIPs have pN/pS values under the upper 1% genome-wide threshold (Additional file 3: Fig. S7b), they do not appear to be functionally decaying. In fact, the number of TIPs at a given gene locus correlates positively with pN/pS, suggesting instead that recurrent visits are functionally relevant and reflect diversifying selection. Consistent with this interpretation, we observed that for a quarter of the loci visited recurrently, the different TE-containing alleles at the locus are associated with gene expression changes in the same direction (Fig. 5a). Congruence is most striking at *FLOWERING LOCUS C* (*FLC*), which encodes a key repressor of flowering and is one of the main genetic determinants of natural variation in the onset of flowering [36]. Specifically, we identified 16 distinct TE-containing *FLC* alleles in total (Fig. 5a), each characterized by a unique insertion within the first intron. This intron is essential to the environmental regulation of *FLC* expression [37] and collectively, the 16 TE insertions are associated with lower expression and earlier flowering (Fig. 5a,c). Together with previous detailed analyses [6, 9], these results indicate that recurrent TE mobilization within *FLC* may be a major contributor of local adaptation.

As expected, genes with multiple TE-containing alleles are strongly depleted in genes associated with core cellular processes, notably translation (Fig. 5d). Instead, genes with increasing numbers of TE-containing alleles are progressively enriched in GO terms linked to defense response, a category of genes under strong diversifying selection [38]. To determine if the recurrence of TE insertions at loci other than *FLC* could also suggest a contribution to local adaptation, we searched for potential associations with environmental differences. We first summarized the 19 WorldClim bio-climatic variables into three climatic envelopes (CEs; Fig. 5e) that together explain > 80% of the climate niche variations observed across the locations of the 1001 Genomes accessions (Additional file 3: Fig. S7d-e). CE1 increases with wetter winters (BIO12 and BIO19) and reduced temperature seasonality (BIO04 and BIO07); CE2 with hotter and drier summers (BIO05 and BIO18) and CE3 with increased temperature changes between winters and summers (BIO04, BIO05 and BIO06). Along each climatic envelope, we then tested for each of the 566 multi-hit gene loci whether the TE-containing alleles are associated with an environmental shift using a logistic GLM that incorporates population structure (see the “Materials and methods” section). In total, 137 gene loci showed significant associations, mainly with CE2 and/or CE3 (Fig. 5f–h). These associations are robust, given that none were identified when the GLM was repeated using random permutations of the environmental variables. Moreover, consistent with the notion that TE-containing alleles of *FLC* are locally adaptive [6, 9], they are found preferentially in parts of the species range characterized by milder winters (low CE3), where they may enable flowering in the absence of vernalization thanks to their lower expression. More generally, TE-containing alleles are systematically at higher frequency when multiple-hit genes show evidence of environmental associations (Fig. 5i), even in the case of exonic TE insertions (Additional file 3: Fig. S7f). Together, these observations suggest that the multiple alleles generated at some loci via recurrent TE insertion are under positive selection [39] and contribute to local adaptation to divergent environments, in line with previous results indicating that repeated loss-of-function mutations are adaptive [40].

Recent transposition tends to be higher among accessions with extreme CE2 values (Fig. 5j), which are enriched for the *NRPE1’* allele (Fig. 5k). To explore the possibility that *NRPE1’* and hence increased TE mobilization are under positive selection in these environments, we quantified haplotype-length decay using iHS and iHH12, two measures used to identify hard and soft sweeps, respectively [41, 42]. Whereas iHS did not reveal any hard sweep, consistent with the wide distribution of the *NRPE1’* allele, iHH12 uncovered marks of soft sweep for accessions located in extreme but not moderate CE2 environments (Fig. 5j; Additional file 3: Fig. S7g). Together, these findings suggest that transposition is a powerful generator of locally adaptive alleles in challenging environments, whose fine tuning by the RdDM machinery is itself the target of natural selection.

### Increased TE mobilization under future climates

**Fig. 6:**
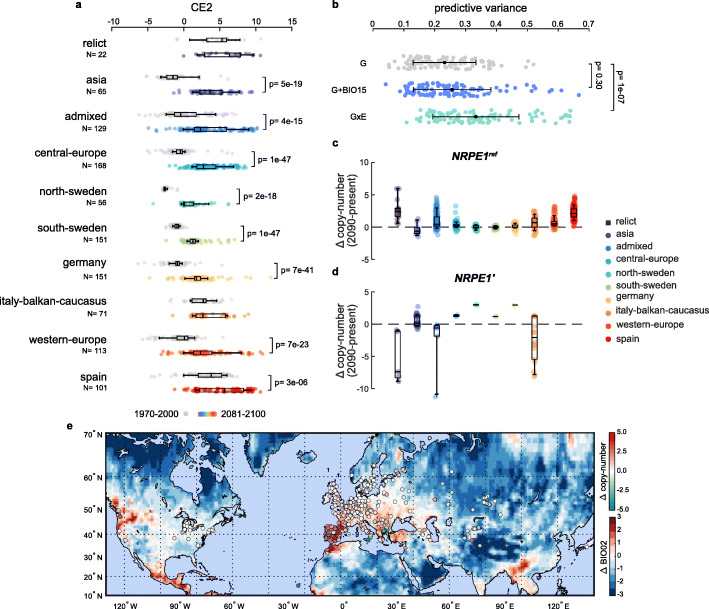
Increased TE mobilization under future climates. **a** Forecasted change of climatic envelope CE2 by genetic group under average CMIP6 SSP5–8.5 GCM for 2081–2100 compared to recent climate (1970–2000). **b** Predictive variance of the numbers of recent copies on 100 random testing sets of 100 accessions for GLMs based on population structure and allelic variation at *NRPE1* (G), including BIO15 (G+BIO15), or with interactions between bio-variables and *NRPE1* (GxE). **c–e** Predicted change by genetic group in copy numbers using GxE GLM with future climate predictions for 2081–2100 for **c** carriers of the reference *NRPE1* allele and **d** carriers of the derived *NRPE1’* alleles. **e** Spatial variations in BIO02 predicted for 2081–2100 and their outcome on copy-number changes by accession

Given the strong environmental sensitivity of TE mobilization, the mutation pressure generated by transposition could be significantly affected by climate change [43]. To investigate this possibility, we first considered forecasts of future climates under the most pessimistic gas emission scenario during the next 60–80 years (CMIP6 SSP5–8.5) for each of the locations occupied by *A. thaliana* accessions of the 1001 Genomes Project. Consistent with the expected global increase in the frequency of hotter and drier summers, CE2 was the most impacted environmental envelope for eight out of the ten genetic groups (Fig. 6a). We then evaluated the predictive power of three GLMs of recent TE mobilization that are based either on genetic variables alone (G), or in combination with the most significant bio-variable (BIO15; G + E) or else with all four major bio-variables together with their GxE interactions (Fig. 3) in 100 random testing sets of 100 accessions each (see the “Materials and methods” section). We confirmed that full GLMs provide the most robust estimates of the number of recent TE insertions (Fig. 6b). Applying the full model, we predict across most locations an increase in transposition activity, which is particularly pronounced in the Mediterranean region (Fig. 6c,e). Nonetheless, because of the GxE interactions involving *NRPE1,* TE mobilization is expected to decrease in Mediterranean populations carrying the *NRPE1’* alleles (Fig. 6d,e), notably in the south of Italy and of the Balkans. Conversely, these alleles should exacerbate transposition at higher latitudes, such as in Sweden. Given that *NRPE1’* appears to be under positive selection in extreme CE2 accessions (Fig. 5j), we anticipate therefore that the GxE interactions involving these derived alleles will play an important role in the survival potential of native populations in the face of climate change.

## Discussion

Understanding how organisms adapt to new environments is of major importance given that climate change is already leading to shifts in species ranges [44]. Standing genetic variation is generally thought to be the main source of rapid adaptation to environmental changes [1] and genomic studies aimed at estimating the evolutionary potential of native populations in future climates have mostly focused on SNPs [45]. Here, we set out to assess the contribution of the rare and typically large-effect alleles created by TE insertions and to determine if transposition activity in nature can ensure a sustained supply of potentially adaptive de novo variants in response to the environment.

### High transposition rate in nature and the evolutionary fate of new TE insertions

Our comprehensive characterization of recent TE mobilization in *A. thaliana* revealed that the natural substitution rate of TE insertions is at least of the same order of magnitude as that of single nucleotides (Fig. 1) and close to the actual transposition rate that we measured experimentally (Figs. 2 and 3). Moreover, given the very stringent filters that we applied throughout our pipeline to detect TIPs, we likely underestimate the mutational pressure exerted by TE mobilization. Our results indicate therefore that TE mobilization is a substantial contributor of new mutations in *A. thaliana*. Furthermore, unlike SNPs which are randomly distributed along the genome and predominantly neutral or mildly deleterious, the most transpositionally active TE families show strong insertion preferences towards genes. As a result, TIPs within or near genes occur at rates similar to that of missense SNPs. However, because TE-containing alleles typically have major functional impacts, they are more rapidly purged by natural selection than nonsense SNPs (Fig. 1; Additional file 3: Fig. S2 and Additional file 3: Fig. S4). Together, these findings indicate that TE mobilization is a major source of large-effect mutations and that sequenced genomes provide mere snapshots of their rapid evolutionary turnover. Given the small genome size of *A. thaliana* (119 Mb) compared to that of most plant species (5.7Gb on average among angiosperms [46]), the generality of the TE dynamics we have uncovered here remains to be determined. Nonetheless, it is worth pointing out that the rate of TE insertion substitutions estimated using cytogenetic analysis of polytenic chromosomes in *D. melanogaster* (0.057 per genome per generation [47];) or based on the detection of structural variants across ~ 15,000 genomes in humans (0.095 per genome per generation [48];), two organisms with widely different genome size and TE composition, is remarkably similar to that we measured in *A. thaliana* (0.06–0.08). This similarity could be fortuitous or suggest instead a form of selection maintaining transposition rates within a relatively close range across eukaryotes.

In spite of the major deleterious effects typically associated with TE insertions within or near genes, we identified over one hundred genes recurrently visited by TEs at the borders of the environmental niche of *A. thaliana* and with signatures of positive selection for the insertion alleles (Fig. 5). The power of transposition to generate adaptive variation is most remarkably illustrated by the multiple independent TE insertions we identified here as well as in previous work [6, 9] at *FLC.* Indeed, these TE-containing *FLC* alleles are predominantly found in accessions from parts of the species range with mild winters, consistent with the major role of this gene in the alignment of flowering time with seasons [36]. More generally, our results suggest that leveraging TE mobilization to generate adaptive allelic variation de novo is faster than relying on introgression of standing genetic variation from distant populations. This scenario stands in sharp contrast with established views on rapid adaptation [1] and repeated evolution [49]. However, most TE-containing alleles under positive selection are at relatively low-frequency, suggesting that they mainly contribute to local micro-evolutionary responses. This pattern is also consistent with observations from catalogs of adaptive loci [50] where large-effect mutations are important contributors of rapid adaptations (e.g. [51]) but not at longer evolutionary timescales, two scenarios reconciling the macro-mutationism of Goldsmith [52] with the infinitesimal model of Fisher [53].

### Modulation of transposition in nature

We identified *NRPE1* as a major genetic determinant of natural transposition. This gene is a key component of the RdDM pathway and the natural allelic series we uncovered includes a truncated form that causes lower CHH methylation and higher levels of transposition at TEs targeted by RdDM (Fig. 2). Previous work indicated that alleles of *CMT2* are also major determinants of CHH methylation variation in *A. thaliana* [28]. However, we failed to detect any association between *CMT2* and recent TE mobilization, either because of insufficient resolution of our GWAS or else because of a more prevalent role of *NRPE1*-dependent CHH methylation in controlling TE mobilization. Although this last hypothesis remains to be tested experimentally, the observation that the *CMT2’* and *NRPE1’* alleles rarely occur together in nature [28] is consistent with this allelic combination being selected against, possibly because a further increase in transposition activity is not sustainable in nature.

Our study revealed that the environment is also an important modulator of transposition activity in nature, which is potentiated further by allelic variation at *NRPE1* (Fig. 3). Moreover, we identified a hardwired network of TFs linked to the environmental responsiveness of several TEs (Fig. 3), including heat-shock factors known to target *ATCOPIA78* (43), the binding of which over regions targeted by RdDM is enhanced in vitro in the absence of DNA methylation (Fig. 3). Also, we note that impaired RdDM is not sufficient in itself to trigger the mobilization of *COPIAs*, which are the main contributors of large-effect genic insertions (Figs. 2 and 4). Indeed, the high transpositional activity associated with the most severe truncation of *NRPE1’* is observed in extreme environments (Fig. 5). Together, these findings suggest that in these environments, *NRPE1’* is akin to mutator alleles described in bacteria. Such alleles are typically favored by selection in harsh environments when the advantage of beneficial mutations is greater than the cost of the higher mutation load they also generate [54–56]. Consistent with this view, *NRPE1’* shows signatures of positive selection in extreme environments (Fig. 5). This last result also supports the notion that evolvability, defined as the ability of organisms to produce adaptive and heritable phenotypic variation, is subject to Darwinian selection [57].

Unlike classical mutator alleles, which are evolutionary transient because of the ever increasing mutation load they generate [58, 59], *NRPE1’* is an ancestrally derived allele that is retained at low frequency across the species range. This long-term retention may be helped by the high selfing rate of *A. thaliana*, which decreases genetic heterogeneity especially at the borders of the species range, thus limiting the speed at which a mutator allele could be lost through outcrossing. Determining the precise conditions that enabled the evolution and persistence of *NRPE1’* will be key to understanding how important such environmentally conditioned mutator alleles might be for adaptation.

### Forecasting transposition in future climates

Our mathematical modeling predicts a major role for transposition in shaping the mutational pressure in changing climates. Indeed, we forecast higher transposition rates in Mediterranean populations in response to global warming (Fig. 6) and thus an accelerated production of large-effect alleles. Some of the new alleles generated in this manner may rescue native *A. thaliana* populations from extinction, notably when they lack advantageous standing variation [60]. However, the increased mutational pressure might also expose populations to a higher risk of extinction by mutational meltdown [61], which is expected to be more important in isolated small populations, where the efficiency of selection is limited. Yet, we showed that mutations generated by TE mobilization typically have strong fitness effects and thus are rapidly purged by natural selection (Fig. 1), consistent with theoretical predictions even for small, selfer populations [61, 62]. Hence, transposition is unlikely to lead to mutational meltdown. Further supporting this notion, we found active TE mobilization in North American accessions, which were introduced on the continent during the seventeenth century from a handful of European individuals [25]. Incidentally, the colonization of North America by this population may help to solve the genetic paradox of invasive species, where despite the lack of genetic variation, colonizing individuals are able to adapt to the very environment they are invading [63, 64]. There, TE mobilization could be seen as a form of genetic bet-hedging strategy where, despite its strongly deleterious effects for a significant fraction of the offspring, it provides unique opportunities to extensively explore the phenotypic landscape and thus reach adaptive optimas in divergent environments.

## Conclusions

We demonstrate that TEs constitute a major and tunable source of large-effect mutations in response to environmental challenges. Our findings as well as modeling provide a first indication that TEs may prevent the demise of native populations at evolutionary risk in the face of climate change, with broad implications for biodiversity.

## Materials and methods

### Detection and filtering of TE insertion polymorphisms (TIPs)

Paired-end short-read whole-genome sequencing data were obtained for 1047 *A. thaliana* accessions from 1001genomes.org and processed using a combined SPLITREADER and TEPID pipeline as described [18]. Briefly, putative non-reference insertion sites detected at the individual level by the SPLITREADER were then intersected and filtered by TE family at the population level in order to merge compatible overlapping insertion sites where at least one individual presented enough supporting reads (DP filter = 3). For both presence and absence variants, local comparisons of the negative coverage were then used to reduce the rate of both false positives and false negatives. Indeed, a drop of coverage in the alignment to the reference genome is expected over true non-reference presence sites compared to surrounding regions (100 bp up and down) and similarly at the edges of true non-reference absence variants. Following this step, high specificity (low rates of false positives) was obtained across TE families, apart from *HELITRON* presence variants (Baduel et al. MMB 2020). Conversely, genomes with little coverage (neither supporting an insertion, i.e., positive coverage, or its absence, i.e., negative coverage) over the insertion site or over the reference TE sequence were classified as NA as they cannot be called by either pipeline. Sites with less than a 100 informative genomes were discarded as these bring little information on the frequency of the TIP across the 1047 genomes. Furthermore, we removed ~ 2500 (2474) non-reference insertion sites where the positive coverage is never higher than the negative coverage within a given carrier, as heterozygous non-reference insertions are not expected in a selfer like *A. thaliana* expect if they occurred in the past one or two generations which could represent transposition events that occurred in the lab. Within TE absence variants, 4455 correspond to fragmented reference TE sequences (4008) or ancestral reference TE sequences also found in the *Arabidopsis lyrata* genome by a BLAST of the 200-bp sequences bridging the two edges of the reference TE sequences (447). These absence calls were also removed as they most likely result from genomic rearrangements produced by unequal crossing-over events or non-homologous recombinations instead of recent TE mobilization events. Although some of absence variants likely reflect excision in non-reference genomes, a significant fraction segregates at frequency > 20% and therefore likely represent recent insertions in Col-0.

### Methylome analysis

Processed bisulfite sequencing (BS-seq) data of 779 of the 1047 genomes was obtained from 1001genomes.org [35]. Methylation files of carriers of the derived *NRPE1’_Δrep* and of the derived *NRPE1’_ΔQS* alleles and 100 carriers of the reference *NRPE1*^*ref*^ allele were merged using the methylpy merge-allc option [65]. Bigwigs were generated using methylpy allc-to-bigwig. Merged bigwigs were then processed and plotted in metaplots over all NRPE1-targeted TEs using deepTools [66] functions computeMatrix and plotProfile. BS-seq data from the experimental *nrpe1* allelic series were obtained from [30] and processed similarly. NRPE1-targeted TEs were defined as overlapping with DMRs identified in the *nrpe1_11* mutant line [30] while CMT2-targeted TEs were defined from *cmt2* DMRs [67].

### Genomic analyses

The SNP vcf file was obtained from 1001genomes.org [26] and genome-wide pairwise divergences were calculated across all pairs of accessions using the allvsall --sample-diff counts-only option of PLINK2 [68] available download at https://www.cog-genomics.org/plink/2.0/. Pairwise SNP differences were then compared to pairwise TIP differences within either only recently diverged accessions (diverging by less than 500 SNPs genome-wide) or 104,700 pairs of all accessions (100 random pairwise comparisons for each accession). A linear regression with no intercept was fit in both cases. The slope of the linear regression calculated over closely related accessions was used to derive the genome-wide TE insertion substitution rate from the one calculated for SNPs (0.2511 per genome per generation; 2.11E-9 per site per generation [25];. For all pairs of accessions, the substitution rate was rescaled to take into account the effect of selection on SNPs which we estimated using the synonymous SNPs, which are expected to be neutral. Indeed, these SNPs are overrepresented relative to all SNPs among distant accessions when compared to their respective proportions among close accessions (Additional file 3: Fig. S2c). We used this discrepancy to estimate the scaling factor of the substitution rate that takes into account the average effect of selection on SNPs (Fig. 1h; Additional file 3: Fig. S2c-e).

Pairwise divergence were calculated within 70-kb windows surrounding each TE insertion site between all carriers of the TE insertion using PLINK2 [68]. The age of TE insertions were then estimated based on the highest pairwise divergence observed within the 70-kb window between any two carriers and divided by the mutation rate (7E-9) [23].

SNPs were annotated using snpEff [69] and sifted by functional effect using snpSift [70] (“ANN [0].EFFECT has ‘synonymous_variant’” for synonymous, “ANN[*].EFFECT has ‘missense_variant’” for missense, “ANN [0].EFFECT has ‘intergenic_region’”, for intergenic, and “ANN[*].EFFECT has ‘stop_gained’” for stop SNPs). Alternate and reference allele SFS for each SNP category were obtained using the --freq command of PLINK2 [68] then folded. The distribution of fitness effects (DFEs) of SNPs and TIPs were calculated from the folded site frequency spectrum (SFS) in 500 bins and compared to synonymous SNPs using DFE-alpha [19] with a two epochs model to take into account the recent population expansion of *A. thaliana* [71]. The time (t2) and the amplitude (n2) of the change of population were set for optimization by likelihood maximization (search_n2 and t2_variable set to 1) starting from the initial t2 value of 50. The mean effect of a deleterious mutation (mean_s) and the shape parameter (beta) of the gamma distribution of the DFE were also set to be optimized by likelihood maximization (mean_s_variable and beta_variable set to 1) starting from the initial values of 0.1 and 0.5 respectively.

Metrics of haplotypic decay (iHS and iHH12) were calculated using selscan [72] by chromosome after phasing biallelic SNPs with missing genotyping rates under 0.2 (plink option --geno) and MAF over 0.001 with shapeit [73]. Chromosomal calculations were then normalized together using selscan’s companion program norm [72].

Estimates of recent TE mobilization were obtained genome-wide or by superfamily using 7436 TIPs segregating at frequencies lower than 0.2% and private or younger than 1000 years old, hereafter referred to as very recent TIPs. In total, 89% of these very recent TIPs were contributed by 77 TE families with more than 20 TIPs species-wide. Genome-wide association study (GWAS) were run using EMMAX [74] using the 845,188 biallelic SNPs with minor allele frequencies > 5% and missing genotyping rate < 10% that have been identified across the 1001 Genomes ([17]; 1001genomes.org) from which was calculated the recommended BN (Balding-Nichols) kinship matrix. Linkage between SNPs were calculated using PLINK [75]. Generalized linear models (GLM) of the combined number of recent TE copies of the 77 most recently mobile TE families were fitted using the MATLAB function fitglm with a Poisson distribution to estimate the percentage of variance explained (PVE) by the explanatory variables provided by the first three principal components (PCs) of the principal component analysis (PCA) of the IBS kinship matrix (which together represent 77.6% of the variation in kinship) with or without the *NRPE1*-*16719082* leading SNP. Including *NRPE1*-*16719082* improved the fit of the GLM to reach 27.5% of PVE compared to only 10.1% with only the three kinship-PCs (Additional file 7: Table S3). For graphical purposes, marginal effects and 95% confidence intervals of each variable in a GLM (Additional file 3: Fig. S3) were represented by approximating the GLM with a linear model and averaging the effect of all the other variables using the MATLAB function plotEffects.

### Plant growth

Seeds from four accessions (Col-0, Tsu-0, Cvi-0 and Shahdara) were grown in a controlled design aimed at propagating successive generations under non-selective conditions (long days). Stratified seeds are first germinated in vitro under standard conditions (½ MS media). At the fully developed cotyledons stage (5 days after sowing, DAS), seedlings are transferred on a new plate containing ½ MS media supplemented with 1% sucrose. After 2 weeks, plants are then transferred to soil in individual pots for setting seeds in a growth room. Five individuals (lines) are randomly selected for the next generation. This was repeated for two successive generations for each accession.

To study the effect of stress on transposition, plants were germinated at 23 °C:19 °C in long days (16 h:8 h light:dark) on ½ MS plates then 2-week-old seedlings were transferred to liquid ½ MS media (0.1% Agar) either pure (control and heat-shock) or containing 1 μM of flagellin (flg-22). After 1 day, heat-shocked seedlings were transferred for 24 h at 6 °C then 24 h at 37 °C then returned to 23 °C:19 °C conditions. After 6 days, all seedlings were transferred to soil and plants were then grown to maturity at 24 °C:22 °C under long days to collect seeds. DNA was extracted from 1000 resulting offspring and subjected to TE sequence capture.

### TE sequence capture

TE sequence capture was performed on exactly 1000 offspring in all cases. Genomic DNA was extracted from seeds using the CTAB method, except in the case of Cvi and plants used in the experiment presented in Fig. 2i, where DNA was extracted from germinated seeds and 10-day-old individuals, respectively. Libraries were prepared using 1 μg of DNA and KAPA HyperPrep Kit (Roche) following manufacturer instructions. Libraries were then amplified through 7 cycles of ligation-mediated PCR using the KAPA HiFi Hot Start Ready Mix and primers AATGATACGGCGACCACCGAGA and CAAGCAGAAGACGGCATACGAG at a final concentration of 2 μM. One microgram of multiplexed libraries was then subjected to TE sequence capture [6, 9]. Enrichment for captured TE sequences was confirmed by qPCR and estimated to be higher than 1000 fold. Pair-end sequencing was performed using one lane of Illumina NextSeq500 and 75-bp reads. Between 15 and 100 million paired reads were sequenced per library. After random downsampling (10 times) to 25 million paired reads of all samples with greater sequencing depth, reads were mapped to the TAIR10 reference genome using Bowtie2 v2.3.25 with the arguments –mp 13 –rdg 8,5 –rfg 8,5 –very-sensitive. An improved version of SPLITREADER (available at https://github.com/baduelp/public) was used to detect new TE insertions. Putative insertions supported by at least two and no more than 15 split-reads and/or discordant-reads at each side of the insertion sites were retained. Insertions spanning centromeric repeats or coordinates spanning the corresponding donor TE sequence were excluded. In addition, putative TE insertions detected in more than one library were excluded to retain only sample-specific TE insertions.

### Environmental associations

Gridded weather and climate data at the 5′ resolution were obtained from WorldClim.org. Current climate for each accession was estimated from 19 bio-climatic variables summarizing monthly averages over the period 1970–2000 (WorldClim version 2.1) on the basis of their GPS coordinates (1001genomes.org) in the 5′ grid. After *z*-scoring, current bio-climatic variables were added sequentially to the GLM of numbers of recent TE copies on the basis of their contribution to the *R*^2^ either as fixed effects or as interaction effects with the *NRPE1*-*16719082* SNP until no added variable increased *R*^2^ by more than 1% in order to prevent hyperinflation of the model (Table S3). For graphical purposes, marginal effects were represented as described above using a linear approximation of the GLM, and conditional effects were estimated for each pair of variables with a significant interaction term using the MATLAB function plotInteractions.

The Mantel test was performed using the MATLAB script RestrictedMantel [76] with 1000 permutations to test for associations between recent TE mobilization for each of the 77 most mobile TE families against each of the 19 current bio-variables after taking into account the IBS kinship matrix. TE families were then clustered by environmental associations using the MATLAB clustergram function based on the correlation distance (one minus the correlation between rows) between their 19 bio-variables association values.

To study the binding potential of transcription factors (TFs) on transposable elements (TEs), we reanalyzed DNA affinity purification and sequencing (DAP-seq) data obtained in *Arabidopsis thaliana* [32] for 529 TFs. We processed this data using a modified version of the bioinformatics pipeline implemented by [32] to consider, in addition to single-mapping reads, reads that map to multiple positions in the genome and that are often associated with identical TE copies present in multiple copies. Single-ended reads were mapped on the TAIR10 genome using Bowtie2 Bv.2.3.2, and PCR duplicates were removed using Picard. The detection of peaks for TF binding was performed with GEM (arguments --k_min 6 --kmax 20 --k_seqs 600 --k_neg_dinu_shuffle --t 5). Density of binding peaks (# peaks / kb) over each TE family were normalized by the genome-wide density of each TF to take into account differences between TFs. Preferential enrichment for a TF binding over a TE cluster was calculated using a Wilcoxon rank sum test of the normalized TF densities over the TE families of a cluster compared to the other recently mobile TE families (out of the 77).

Raw RNA-seq data across environmental conditions were obtained from publicly available datasets (Additional file 9: Table S4). Expression level was calculated by mapping reads using STAR v2.5.3a [77] on the *A. thaliana* reference genome (TAIR10) with the following arguments –outFilterMultimapNmax 50 --outFilterMatchNmin 30 --alignSJoverhangMin 3 --alignIntronMax 10000. Duplicated pairs were removed using picard MarkDuplicates. Read counts were calculated over annotated genes and TE sequence features and normalized between samples using DESeq2 [78].

Ecological niche modeling of the 1047 accessions was performed by PCA of the 19 bio-climatic variables which were summarized into three climatic envelopes (CE1–3) which together explained 79.9% of the environmental variance. Association between the presence of a TE insertion within or near (250 bp) recurrently hit genes (566 with 3 or more TIPs) and the three climatic envelopes (CE1–3) was calculated using a binomial GLM (logit link function) using MATLAB fitglm function. *P* values were then corrected using the Benjamini and Hochberg correction for false discovery rate (FDR) using MATLAB fdr_bh function. Random expectations were calculated by shuffling randomly the environment of all the accessions.

Random expectations of the number of genes or pseudogenes with TIPs located within 250 bp were calculated by randomly distributing 23,331 TIPs across the genome. The average and standard deviation in the number of genes with random TIPs nearby were calculated over tenreplicates of the random distribution of TIPs.

### Forecasting TE mobilization

Future climate forecasts were obtained by averaging CMIP6 downscaled future climate projections (calibrated on WorldClim v2.1 as baseline) for the 2081–2100 period with the most extreme Shared Socio-economic Pathway (SSP) 585 under four global climate models (GCMs): CNRM-CM6-1, IPSL-CM6A-LR, MIROC6, and MRI-ESM 2-0 to take into account the heterogeneity between different models. Future bio-variable values for each accession were then *z*-scored based on the mean and standard deviation of the current climate bio-variables in order to use them as inputs in the GLMs trained using current climate and estimate the future TE mobilization predicted by the model. Our model assumed that genetic structure will remain unchanged over this short evolutionary time. To evaluate the predictive power of the model, we extracted 100 random accessions (~ 10% testing set) and estimated the parameters of the full GLM (Table S2) using the remaining 947 accessions (training set). Using these parameters, we then compared the number of recent TIPs predicted by the GLM for the 100 accessions of the testing set against the recent TIPs observed in these genomes and repeated the random sampling of a testing set a 100 times (Fig. 6). For each accession, we thus obtained ~ 10 estimates of the predicted number of recent TIPs from which we could derive a predictive accuracy (standard deviation) and evaluate the prediction behavior (over or underestimating) using a linear regression (Additional file 3: Fig. S8). We considered as outliers the five accessions for which differences between the expected number of recent TE insertions deviated from the observed by more than four standard deviations (*P* < 0.0001), which we removed from further predictive analyses.

### Statistical analyses

All statistical analyses and graphics were realized using MATLAB R2020a, The MathWorks, Natick, 2020.

## Supplementary Information

**Additional file 1: Table S1.** List and information of 1047 *A. thaliana* accessions included in the study.

**Additional file 2: Dataset S1.** List of 23,331 TIPs and their presence (1) or absence (0) across 1047 accessions.

**Additional file 3: Fig. S1-S8.**

**Additional file 4: Table S2.** Results of generalized linear models of recent transposition based on genetic factors.

**Additional file 5: Dataset S2.** List of 154 transposition events identified in 1000 offspring of WT and nrpd1 parents of the Col-0 accession grown under standard conditions and their presence (1) or absence (0) across both samples.

**Additional file 6: Dataset S3.** List of 331 transposition events identified in 1000 offspring the Cvi-0, Sha-0, Tsu-0 and Col-0 accessions derived from parents grown under standard conditions grown under standard conditions and their presence (1) or absence (0) across samples.

**Additional file 7: Table S3.** Results of generalized linear models of recent transposition based on genetic and environmental factors.

**Additional file 8: Dataset S4.** List of 189 transposition events identified in 1000 offspring of Col-0 WT and nrpd1 parents grown under standard conditions or exposed to heat-shock or flagellin and their presence (1) or absence (0) across both samples.

**Additional file 9: Table S4.** List of publicly available RNA-seq datasets used for transcriptomic analyses.

**Additional file 10.** Review history.

## Data Availability

The TE sequence capture datasets generated and analyzed during the current study are available in the European Nucleotide Archive (ENA) under project PRJEB43262 [79]. WGS data was obtained in FASTQ format for the 1047 accessions of the 1001genomes.org project from NCBI SRA Project PRJNA273563 [80]. Normalized transcriptome data (RNA-seq) was obtained from NCBI GEO Accession GSE80744 [81]. Processed BS-seq data was obtained from NCBI GEO Accession GSE43857 [82]. Raw DAP-seq data was obtained from NCBI GEO Accession GSE60143 [83]. Raw RNA-seq data across environmental conditions were obtained from the publicly available sources listed in Additional file 9: Table S4.
