# Supplementary material for: Genetic and environmental modulation of transposition shapes the evolutionary potential of Arabidopsis thaliana
Source: Genome Biol. 2021 May 6;22:138. doi: 10.1186/s13059-021-02348-5 (PMC8101250; doi:10.1186/s13059-021-02348-5)
Supplement: Supplementary file 3 — Additional file 3: Fig. S1-S8. [file 13059_2021_2348_MOESM3_ESM.pdf]

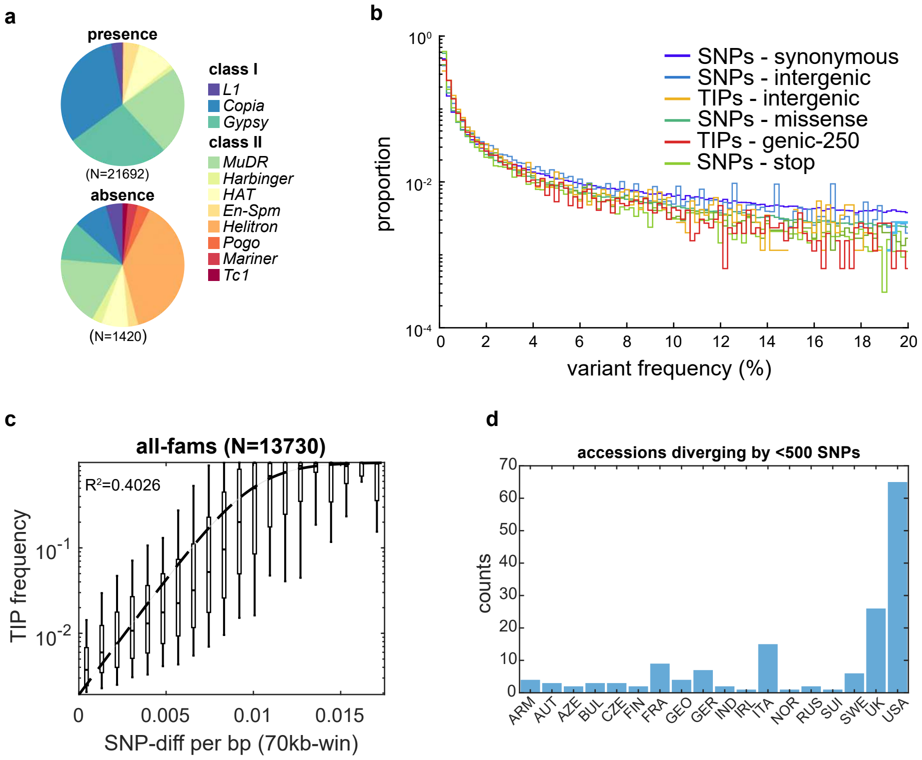

Figure S1. (a) Contribution by TE superfamily to TE presence variants and TE absence variants. (b) Folded low-frequency spectrum (<20%) of TIPs further than 2kb from the nearest gene (intergenic), within 250bp of the nearest gene (genic-250) and bi-allelic SNPs by functional category (synonymous, intergenic, missense, and stop variants). (c) Correlation between TIP frequency and maximum pairwise SNP differences observed within 70kb between any two carriers for all non-private TIPs. (d) Distribution of closely related accessions by genetic group.

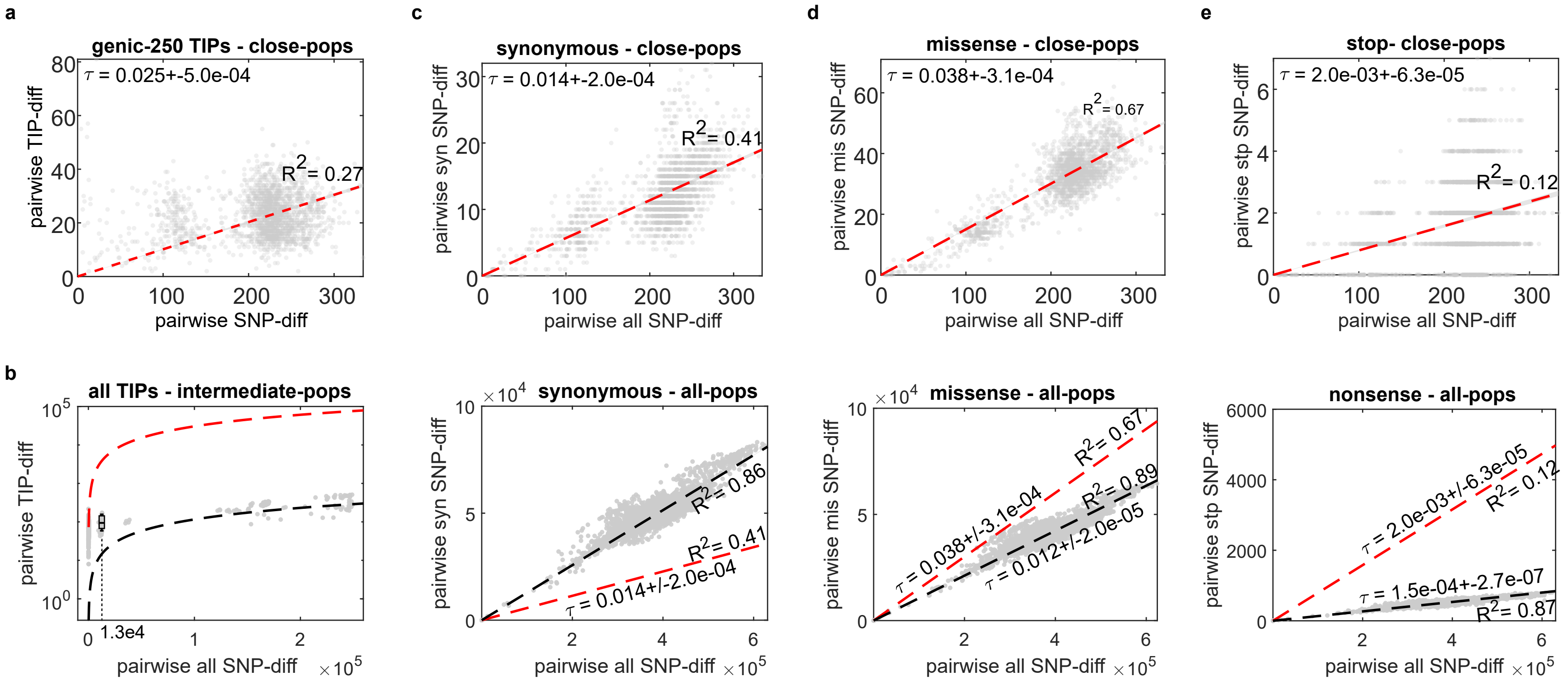

Figure S2. (a) Pairwise differences in TIPs within and near genes (<250bp) and SNPs for all accessions diverging by <500 SNPs. Regression line and confidence intervals are indicated in red and gray, respectively. The substitution rate of this category of TIPs calculated from the substitution rate of 2.11e-9 per site per generation measured by Exposito-Alonso et al. [25] scaled by the regression slope is indicated with its 95% confidence intervals. (b) Pairwise differences in TIPs and SNPs between accessions. Regression lines between all and closely related accessions are shown in black and red, respectively. Boxplot of pairwise TIPs differences observed within 116 pairs of intermediate accessions (diverging by 10,000 to 15,000, on average 12,619 SNPs genome wide, i.e. ~50,000 generations). Mean TIPs pairwise differences in these accessions represents 2.75% of the expectation based on closely related accessions. (c-e) Pairwise differences in respectively synonymous, missense, and nonsense SNPs versus all SNPs between close (top) and all (bottom) accessions. Regression lines between all and closely related accessions are shown in black and red, respectively. Substitution rates for each category of SNPs calculated from the regression slopes and the substitution rate of all SNPs are indicated with their 95% confidence intervals. For closely related accessions we used the substitution rate of 2.11e-9 per site per generation measured by Exposito-Alonso et al. [25]. For all accessions, we rescaled this substitution rate to take into account the effect of natural selection estimated from the putatively neutral synonymous SNPs (see Methods).

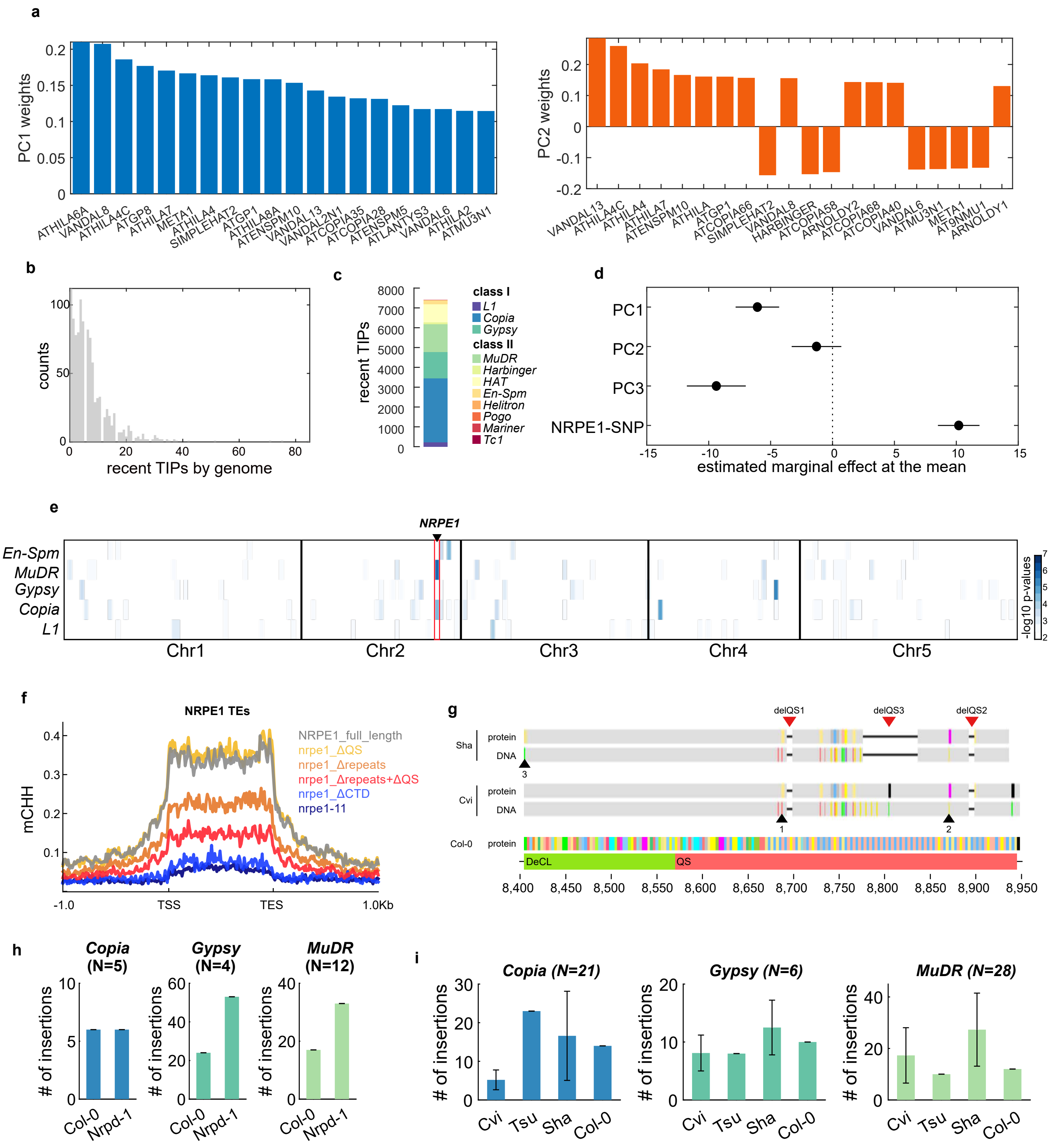

Figure S3. (a) Weights of first 20 TE families in PC1 and PC2 of PCA of recent TE mobilization (TIP frequency <=5%, LF5) (b) Variations in numbers of very recent TIPs (frequency <=0.2% and private or <1,000 years old) across 1047 accessions (c) Contribution to very recent TIPs by superfamily. (d) Estimated marginal effect at the mean of PC1-2-3 of the kinship matrix and the NRPE1 allele in GLM of genome-wide very recent TE mobilization. (e) Heatmap of top percentile -log10 p-values in 500kb windows across the 5 chromosomes from the GWAS of very recent TE mobilization by superfamily against MAF005 SNPs. (f) Metaplot of CHH methylation over NRPE1-TEs in NRPE1 mutant constructs from Wendte et al. 2017. (g) Detailed alignment of DNA and protein sequences of the last exon of NRPE1 in Col-0, Sha [29], and Cvi. The three deletions in the QS domains (6bp at 2:16723152, 60bp at 2:16723235, and 9bp at 2:16723351) are indicated with red arrows and their closest tagging SNPs with black arrows and corresponding numbers. (h) Transposition rates of the three most mobile superfamilies (COPIA, GYPSY, and MuDR) in 1,000 offspring of WT or nrpd1 Col-0 parents grown under standard conditions. (i) Transposition rates of the three most mobile superfamilies (COPIA, GYPSY, and MuDR) in 1,000 offspring of Cvi, Tsu, Sha, and Col-0 parents grown under standard conditions

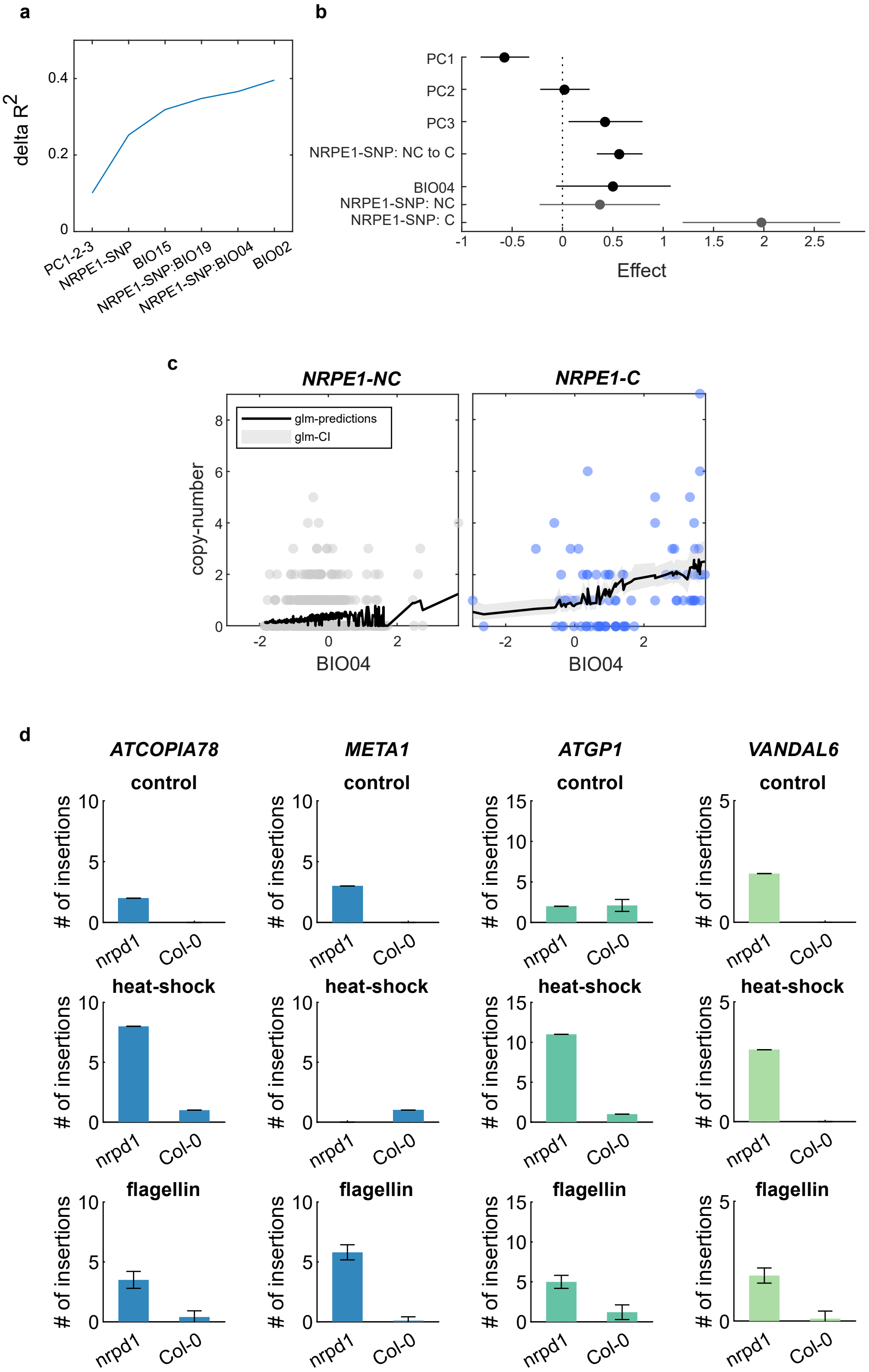

Figure S4. (a) Cumulative fraction of variance explained (R2) by successive addition of PC1-2-3, NRPE1 allele, and BIO02, BIO04, BIO15, BIO19 in GLM with interaction effects of very recent transposition. (b) Marginal effect at the mean of PC1-2-3, NRPE1 allele, and BIO04 and estimated interaction effect between BIO04 and NRPE1 in GLM of very recent ATCOPIA78 transposition. (c) Scatter plot of very recent ATCOPIA78 transposition against BIO04 (left) and BIO19 (right) in non-carriers (NC, up) and carriers (C, down) of derived NRPE1' allele. GLM predictions and confidence-intervals are indicated in black and grey, respectively. (d) Numbers of new insertions by TE family detected in 1,000 offspring of WT and nrpd1 Col-0 parents and WT Ler parents grown under standard conditions or exposed to heat-shock or flagellin.

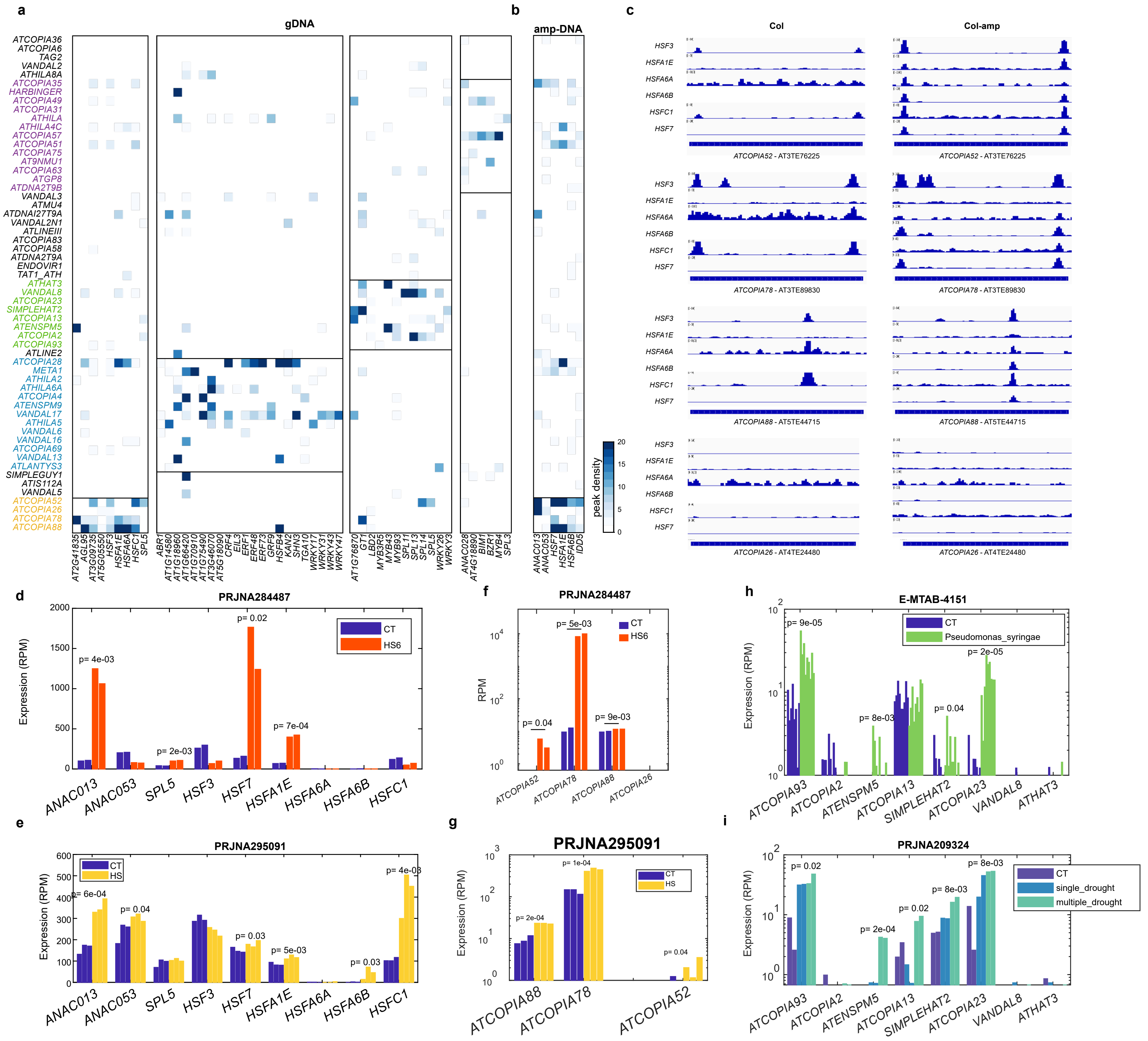

Figure S5. (a) Normalized peak density of in-vitro binding of TFs (DAP-seq) enriched over each of the four TE clusters identified in Col-0 gDNA and (b) over the “temperature” TE cluster in Col-0 PCR-amplified DNA (c) Tracks of DAP-seq peaks of heat-shock factors HSF3, HSFA1E, HSFA6A, HSFA6B, HSFC1, and HSF7 in Col-0 gDNA and PCR-amplified DNA over the four TEs composing the “temperature” cluster. (d-e) Normalized RNA-seq expression levels of TFs enriched over the “temperature” TE cluster in both Col-0 gDNA and PCR-amplified DNA under two heat-shock experiments. (f-g) Normalized RNA-seq expression levels of the four TEs composing the “temperature” TE cluster under two heat-shock experiments. (h-i) Normalized RNA-seq expression levels of the TEs composing one “precipitation” TE cluster under exposure to biotic stress (P. syringae) or drought.

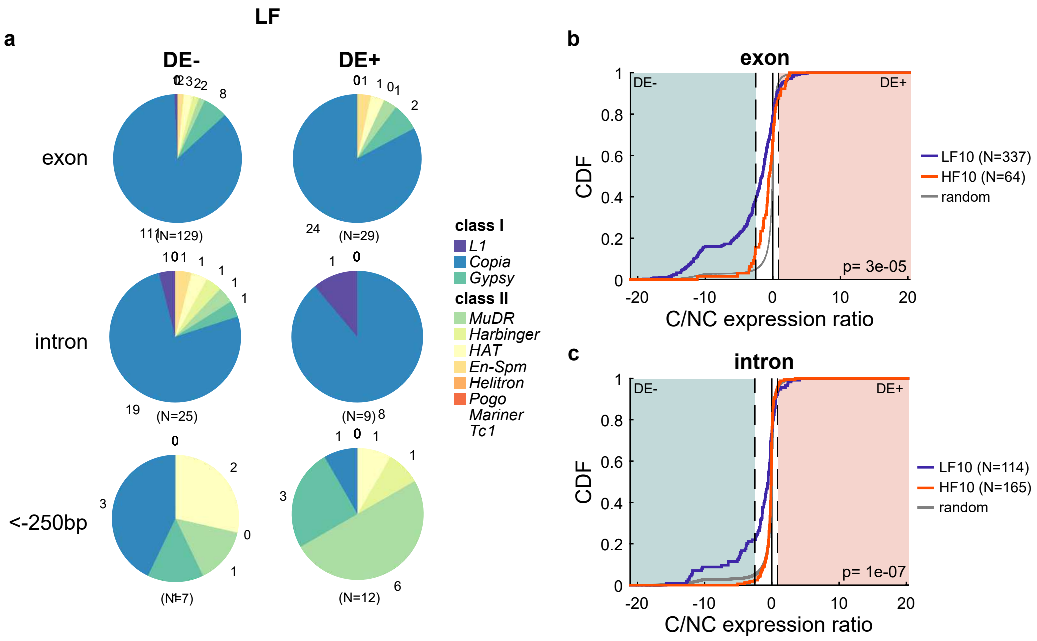

Figure S6. (a) Contribution by TE superfamily to TE presence variants with negative (DE-) and positive (DE+) transcriptomic impacts in exons, introns, and close promoter (<-250bp).(b-c) Distribution of transcriptomic impacts (C over NC log ratios) for exonic and intronic TE presence variants at low-frequency (LF) vs high-frequency (HF) compared to random sampling of carriers and non-carriers. Dashed lines indicate top and bottom 5% values of random distribution, under and over which C/NC ratios are considered extreme.

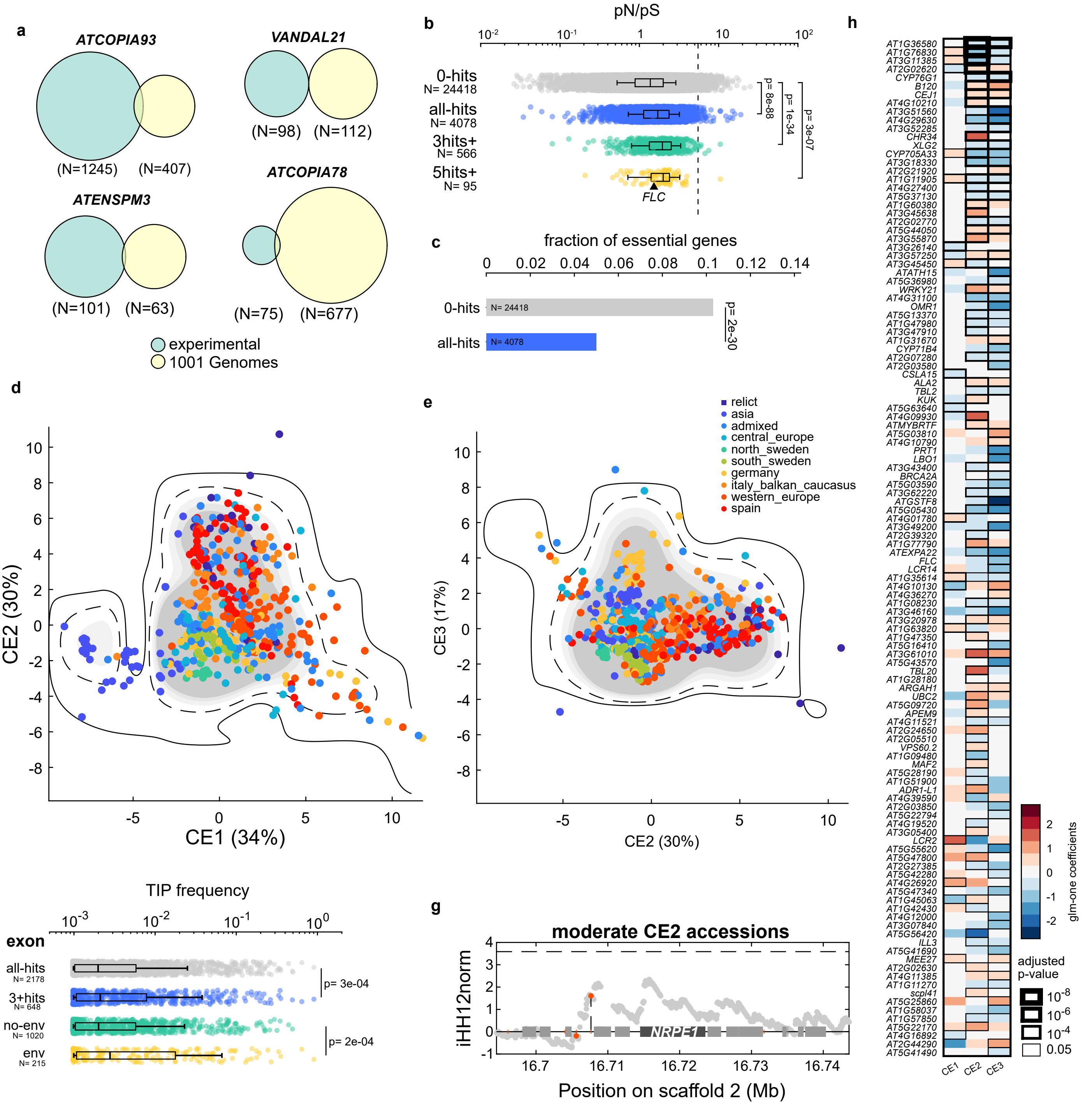

Figure S7. (a) Genes visited within 250bp by ATCOPIA93, ATCOPIA78, VANDAL21, and ATENSPM3 in experimental transposition accumulation lines [9] compared to that found in natural accessions. (b) Ratio of non-synonymous (missense, pN) against synonymous SNPs (pS) for genes never visited by TE insertions (0 hits), visited at least once (all-hits), at least thrice (3hits+), and 5 times or more (5hits+). P-values of Wilcoxon test between distributions are indicated (c) Proportion of essential genes found within each category. P-values of Fisher exact tests between categories are indicated. (d-e) First three climatic envelopes (CE1-3) from principal component analysis of 19 BIO variables across 1047 accessions. (f) Frequency of TE insertions found within exons of genes visited at least once (all-hits), 3 times or more (3hits+), in association with a CE shift (env) or not (no-env). The p-values of Wilcoxon tests between distributions are indicated. (g) iHH12 values in moderate CE2 accessions (two middle quartiles) across the NRPE1 region with in black indicated values above the genome-wide 1% threshold (dashed line). (h) Heatmap of associations in logistic GLM between presence of TE insertion within or near genes and the three climatic envelopes (CE1-3).

a

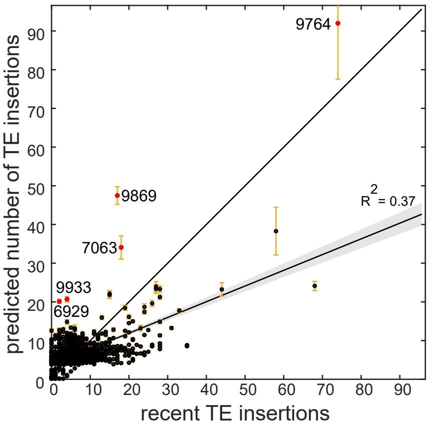

Figure S8. (a) Average number of TE insertions predicted by accessions of 100 randomly sampled testing sets of 100 accessions of GLMs based on GxE interaction terms with coefficients trained over the remaining 947 accessions. Error bars represent standard error across predictions and shaded area represent 95% confidence intervals around linear regression. The five accessions showing large deviations between the predicted and observed number of TE insertions, and therefore excluded from our forecast models, are indicated in red.
