# Supplementary material for: Genetic and environmental modulation of transposition shapes the evolutionary potential of Arabidopsis thaliana
Source: Genome Biol. 2021 May 6;22:138. doi: 10.1186/s13059-021-02348-5 (PMC8101250; doi:10.1186/s13059-021-02348-5)
Supplement: Supplementary file 10 — Additional file 10. Review history. [file 13059_2021_2348_MOESM10_ESM.docx]

Review History

**First round of review**

**Reviewer 1**

**Are you able to assess all statistics in the manuscript, including the appropriateness of statistical tests used?**

No.

**Comments to author:**

The manuscript by Baduel and colleagues reports on the analysis of transposon insertion polymorphisms (TIPs) in over 1000 Arabidopsis genomes in order to evaluate the adaptive role of TIPs in this species. The authors confirm their previous finding that FLC is a hotspot of TIPs and further correlate bioclimatic parameters to the distribution of very recent TIPs. They reveal the role of a natural variant of NRPE1. Finally they propose a model to predict the impact of climate change on TIPs. By combining in a very elegant way genomics and climatic data together with modeling, this excellent study reveals the high turnover of TIPs in a model species and its control by both epigenetic and climatic factors.

The data are clearly exposed and the figures are very well designed. Here are a few comments:

1) From their data the authors conclude that the natural rate of TE insertion is of the same order than SNPs (Page27 Line8): it should be noted that these conclusions relate to this species with a small TE compartment (medium size plant genome is over 1Gb). P23 L32: "is at least of the same order": As mentioned by the autohrs this rate is likely underestimated (because of short reads) so the authors might rephrase to "at least" of the same order.

2) While ATCOPIA78 and ATCOPIA93 might seem familiar TE names to people working on Arabidopsis, they are mainly known as ONSEN and EVD, owing to their specific regulation (Mirouze et al Nature 2009, Ito et al Nature 2012). As this paper is expected to have a high impact and be of interest for a broad audience these short names should be cited for clarity.

3) P24 L13 FLC has already been identified by the same authors as a hotspot of TE insertions and this could be highlighted here.

4) P24 L24 Rey et al Trends in Ecology and Evolution 2016 could be cited as they proposed a role for TEs in rapid adaptation.

5) P13 L33 How do these results compare with the previous studies on HSF bindig sites on ONSEN for instance? (see Cavrak et al PLoS Genetics 2014; Pietzenuk et al, Genome Biology 2016)?

6) Transposition rate is calculated for 1000 seeds. Why do the authors refer to F1 seeds in the legend (if from WT for instance). F1 refers to a genetic cross, is this the case here? Moreover, this rate seems to vary for WT plants (0.05 in Fig2i vs 0.01 in Fig3f), which is not so surprising. Were the experiments reproduced with the same trend?

7) Figure 6 d and e are not described in the results. The green dots on Figure 6e are worth describing, does the model predict a genomic purge of TEs in some cases?

Minor comments:

P26 line 35-40 a couple of typos

Figure 6: typo in the legend G+E replaced by G+BIO15

FigS8: the rationale for excluding the 5 outliers could be clarified.

**Reviewer 2**

**Are you able to assess all statistics in the manuscript, including the appropriateness of statistical tests used?**

No.

**Comments to author:**

the manuscript submitted by baduel et al. is a report of a comparative genomic analaysis of the 1000 arabidopsis genomes focusing on transposable elements.

This paper is well written with clear objectives and a comprehensive amount of analyses that make the conclusions drawn by the authors quite convincing. I think that this paper deserves publication in GB. I've got only few suggestions that may improve its quality.

- the fact that TIPs are found in low frequency suggest that they are recent and/or selected against because of negative effects on fitness. IMO, the authors phrase this incorrectly : "TE mobilization mainly generates highly deleterious genic mutations" would suggest that TIPs within genes are being eliminated. But these TIPs have been identified in accessions that were collected in natural populations as seeds. This suggests that the individuals harboring these TIPs were fertile. Moreover, these accessions are now distributed among the Arabidopsis community and I don't think that there is any problem with their propagation. This leads me to ask the authors to temper their claim. "highly deleterious genic mutations" is not appropriate, but the transcriptome data provided in the paper is very convincing : insertions impede gene expression. With such good quality data in their hands, the authors should discuss this point a bit further. These TIPs may decrease fitness in the wild, but as they probably know, the measure of fitness in natura is probably one of the hardest parameter to evaluate. This raises the question whether there is structural or functional redundancy for the mutated genes.

- As a non Arabidopsis specialist, I've read about Evade and Onsen retrotransposons. I can't remember AtcopiaXXX nomenclature. I am pretty sure I have already written this in a previous review of a manuscript from this group. TE nomenclature is a never ending discussion, but once one names a particular family because of a particular feature, it could be so easy to accept it (minor comment...)

- I very much liked the bulk seedling sequencing of both nrpd mutant and natural accessions progenies. This provides a convincing result regarding transpositionnal activity. However, comparing the mutant with Sha accession is not 100% relevant because of the difference in genetic background. The trend may be there, but maybe could the authors temper their conclusions a bit.

- "Increased TE mobilization under future climates".....This may be a good work package for a grant proposal, but as it stands, the data reported here on arabidopsis could eventually be relevant for Arabidopsis populations [which represent (no offense) a negligible portion of global plant biomass !]. I think this should be tested on other plant species with a genome which is more representative of the plant kingdom [angiosperm genome size has a mode of 800 Mb and an average of 1.5 Gbp].

-Last but not least : Could the authors comment on the fact that their GWAS did not give the same result as in their Elife paper (2016) based on 200 accessions. What about the association with met2 gene ???

**Authors Response**

**Point-by-point responses to the reviewers’ comments:**

**Reviewer 1:**

The manuscript by Baduel and colleagues reports on the analysis of transposon insertion polymorphisms (TIPs) in over 1000 Arabidopsis genomes in order to evaluate the adaptive role of TIPs in this species. The authors confirm their previous finding that FLC is a hotspot of TIPs and further correlate bioclimatic parameters to the distribution of very recent TIPs. They reveal the role of a natural variant of NRPE1. Finally they propose a model to predict the impact of climate change on TIPs. By combining in a very elegant way genomics and climatic data together with modeling, this excellent study reveals the high turnover of TIPs in a model species and its control by both epigenetic and climatic factors.

*We thank reviewer #1 for their enthusiastic comments.*

The data are clearly exposed and the figures are very well designed. Here are a few comments:

1) From their data the authors conclude that the natural rate of TE insertion is of the same order than SNPs (Page27 Line8): it should be noted that these conclusions relate to this species with a small TE compartment (medium size plant genome is over 1Gb). P23 L32: "is at least of the same order": As mentioned by the autohrs this rate is likely underestimated (because of short reads) so the authors might rephrase to "at least" of the same order.

*We have now included a discussion of this point (p25 l24 of the revised manuscript: “Given the small genome size of A. thaliana (119Mb) compared to that of most plant species (5.7Gb on average among angiosperms (47)), the generality of the TE dynamics we have uncovered here remains to be determined. Nonetheless, it is worth pointing out that the rate of TE insertion substitutions estimated using cytogenetic analysis of polytenic chromosomes in D. melanogaster (0.057 per genome per generation; [48]) or based on the detection of structural variants across ~15,000 genomes in humans (0.095 per genome per generation; [49]), two organisms with widely different genome size and TE composition, are remarkably similar to that we measured in A. thaliana (0.06-0.08). This similarity could be fortuitous or suggest instead a form of selection maintaining transposition rates within a relatively close range across eukaryotes.”*

2) While ATCOPIA78 and ATCOPIA93 might seem familiar TE names to people working on Arabidopsis, they are mainly known as ONSEN and EVD, owing to their specific regulation (Mirouze et al Nature 2009, Ito et al Nature 2012). As this paper is expected to have a high impact and be of interest for a broad audience these short names should be cited for clarity.

*We have included in the revised version of the manuscript the names ONSEN and EVADE whenever we refer respectively to the single mobile copies of ATCOPIA78 and ATCOPIA93 that are present in the reference genome. However, we have kept the family name whenever we refer to transposition activity in nature, as our approach does not enable us to identify the family member(s) involved.*

3) P24 L13 FLC has already been identified by the same authors as a hotspot of TE insertions and this could be highlighted here.

*We now mention p26 l16 the references of Quadrana et al. 2016 and Quadrana et al. 2019 where a subset of the TE insertions at FLC were previously identified and studied in greater depth.*

4) P24 L24 Rey et al Trends in Ecology and Evolution 2016 could be cited as they proposed a role for TEs in rapid adaptation.

*We have now included this reference p22 l22.*

5) P13 L33 How do these results compare with the previous studies on HSF bindig sites on ONSEN for instance? (see Cavrak et al PLoS Genetics 2014; Pietzenuk et al, Genome Biology 2016)?

*We added further mentions to the studies of Cavrak et al. and Pietzenuk et al. as the consistencies with our results are indeed noticeable, particularly the importance of HSFA1s in the heat-shock response of ONSEN (Cavrak et al.) and the heat sensitivity of ATCOPIA28 (Pietzenuk et al. 2016), which we found to share binding sites of HSFs (Fig. 5g). We have also indicated on Fig. 5h the locations of the tandem of heat-responsive elements (HREs) identified by Cavrak et al 2014 on the LTRs of ONSEN.*

6) Transposition rate is calculated for 1000 seeds. Why do the authors refer to F1 seeds in the legend (if from WT for instance). F1 refers to a genetic cross, is this the case here? Moreover, this rate seems to vary for WT plants (0.05 in Fig2i vs 0.01 in Fig3f), which is not so surprising. Were the experiments reproduced with the same trend?

*We have removed the incorrect labelling of seeds as “F1” in the revised version of the manuscript and replaced with “offspring”. We indeed observe variations in the transposition rate between our Col-0 WT samples, which likely results from slight differences in growth conditions that have been used across experiments. Incidentally, this transposition rate variation also reflects the extreme environmental sensitivity of transposition that we describe. We discuss these observations in the revised version of the manuscript (p12 ll 14-17).*

7) Figure 6 d and e are not described in the results. The green dots on Figure 6e are worth describing, does the model predict a genomic purge of TEs in some cases?

*Thank you for pointing out this omission. We have added the missing references to Fig. 6d and 6e as well as highlighted the strong reduction of TE mobilization in the Italian and south Balkan accessions. As a result, the mutation pressure is expected to be reduced in these genomes due to reduced transposition rates not from increased purge of TE insertions.*

Minor comments:

P26 line 35-40 a couple of typos

*corrected.*

Figure 6: typo in the legend G+E replaced by G+BIO15

*corrected.*

FigS8: the rationale for excluding the 5 outliers could be clarified.

*We have further developed our rationale p38 l5 to clarify.*

**Reviewer 2:**

the manuscript submitted by baduel et al. is a report of a comparative genomic analaysis of the 1000 arabidopsis genomes focusing on transposable elements.

This paper is well written with clear objectives and a comprehensive amount of analyses that make the conclusions drawn by the authors quite convincing. I think that this paper deserves publication in GB. I've got only few suggestions that may improve its quality.

*We thank reviewer #2 for their positive comments.*

- the fact that TIPs are found in low frequency suggest that they are recent and/or selected against because of negative effects on fitness. IMO, the authors phrase this incorrectly : "TE mobilization mainly generates highly deleterious genic mutations" would suggest that TIPs within genes are being eliminated. But these TIPs have been identified in accessions that were collected in natural populations as seeds. This suggests that the individuals harboring these TIPs were fertile. Moreover, these accessions are now distributed among the Arabidopsis community and I don't think that there is any problem with their propagation. This leads me to ask the authors to temper their claim. "highly deleterious genic mutations" is not appropriate, but the transcriptome data provided in the paper is very convincing : insertions impede gene expression. With such good quality data in their hands, the authors should discuss this point a bit further. These TIPs may decrease fitness in the wild, but as they probably know, the measure of fitness in natura is probably one of the hardest parameter to evaluate. This raises the question whether there is structural or functional redundancy for the mutated genes.

*We agree with reviewer #2 that the TIPs we identify in this study are viable and do not lead to major developmental defects which would have prevented the collection and propagation of these accessions in the lab and eventually the sequencing of their genomes. As a result, we are indeed blind to the most deleterious mutations generated by TE insertions, yet we still observe that the rare TE insertions that we do observe are strongly purged by natural selection, consistent with their often major transcriptomic effects. Furthermore, and as reviewer #2 points out, fitness in the wild is very difficult to measure as it depends on many parameters poorly represented by culture conditions in the lab. Thus, the fact that accessions carrying these TE insertions can easily be propagated in the lab cannot be taken as an indication that they have little fitness effect in the wild. Therefore, we maintain that, based on the evidence in our hands, most of the rare TE insertion events we have identified within or near genes have strong deleterious effects.*

- As a non Arabidopsis specialist, I've read about Evade and Onsen retrotransposons. I can't remember AtcopiaXXX nomenclature. I am pretty sure I have already written this in a previous review of a manuscript from this group. TE nomenclature is a never ending discussion, but once one names a particular family because of a particular feature, it could be so easy to accept it (minor comment...)

*Following a similar comment from reviewer #1, we have added the ÉVADÉ (EVD) and ONSEN denominations wherever appropriate (see our response to reviewer #1).*

- I very much liked the bulk seedling sequencing of both nrpd mutant and natural accessions progenies. This provides a convincing result regarding transpositionnal activity. However, comparing the mutant with Sha accession is not 100% relevant because of the difference in genetic background. The trend may be there, but maybe could the authors temper their conclusions a bit.

*We thank the reviewer for raising their concern about this statement. We have modified this statement to make explicit that differences in genetic backgrounds may also contribute to the variation in transposition rate we observed among accessions and rephrased our conclusion to state that ”This observation is consistent with NRPE1’ causing increased TE mobilization.” (p9 l14-15).*

- "Increased TE mobilization under future climates".....This may be a good work package for a grant proposal, but as it stands, the data reported here on arabidopsis could eventually be relevant for Arabidopsis populations [which represent (no offense) a negligible portion of global plant biomass !]. I think this should be tested on other plant species with a genome which is more representative of the plant kingdom [angiosperm genome size has a mode of 800 Mb and an average of 1.5 Gbp].

*Following a similar comment from reviewer #1 we have added a note (pp25-26) to raise the possibility that the picture we obtained in A. thaliana may not be generalizable to much larger plant genomes (see response to reviewer #1).*

-Last but not least : Could the authors comment on the fact that their GWAS did not give the same result as in their Elife paper (2016) based on 200 accessions. What about the association with met2 gene ???

*The focus of our previous work (Quadrana et al 2016) was to identify genetic factors associated with copy number variation at the single TE family level, not global transposition activity across all TE families. We now mention this previous study (p7 line 24) to contrast it with the one presented here.*

**Second round of review**

**Reviewer 1:**

I thank the authors for their excellent work in answering reviewers' concerns.

One point of detail I would like to raise for clarity concerns the nomenclature of TEs. ONS and COPIA78 are actually synonymous, indeed in the TE community the automatic name generated during genome annotation are commonly replaced with a nick name (easier to remember) when this family is further characterized. Of note this is also true with genes and nobody refers to AT5G66750 for instance! No name is given to a TE copy or locus as it would not make sense. Probably Dr Voinnet introduced some confusion when naming one copy of EVD/COPIA93 ATRAPE, but he was then a newbie in the field of TEs..

The paper as it is will make a great contribution to the field and is likely to be massively cited.
